# Endogenous TDP-43 mislocalization in a novel knock-in mouse model reveals DNA repair impairment, inflammation, and neuronal senescence

**DOI:** 10.1186/s40478-025-01962-9

**Published:** 2025-03-08

**Authors:** Joy Mitra, Manohar Kodavati, Prakash Dharmalingam, Erika N. Guerrero, K. S. Rao, Ralph M. Garruto, Muralidhar L. Hegde

**Affiliations:** 1 Division of DNA Repair Research, Center for Neuroregeneration, Department of Neurosurgery, Houston Methodist Research Institute, Houston, TX 77030 USA; 2 Department of Biotechnology, Koneru Lakshmaiah Education Foundation Deemed to Be University, Green Fields, Vaddeswaram, Andhra Pradesh 522502 India; 3 Department of Anthropology, Binghamton University, State University of New York, Binghamton, NY 13902 USA; 4 Department of Biological Sciences, Binghamton University, State University of New York, Binghamton, NY 13902 USA; 5 Department of Neuroscience, Weill Cornell Medical College, New York, NY 10065 USA; 6 Present Address: Gorgas Memorial Institute for Health Studies, Avenida Justo Arosemena y Calle 35, Panama City, Republic of Panama; 7 Present Address: Sistema Nacional de Investigación, SENACYT, Panama City, Republic of Panama

**Keywords:** Amyotrophic lateral sclerosis, TDP-43, Inflammation, DNA damage, Senescence, Motor deficits, Muscle atrophy, Neurodegeneration, Motor neuron

## Abstract

TDP-43 mislocalization and aggregation are key pathological features of amyotrophic lateral sclerosis (ALS)- and frontotemporal dementia (FTD). However, existing transgenic hTDP-43 WT or ∆NLS-overexpression animal models primarily focus on late-stage TDP-43 proteinopathy. To complement these models and to study the early-stage motor neuron-specific pathology during pre-symptomatic phases of disease progression, we generated a new endogenous knock-in (KI) mouse model using a combination of CRISPR/Cas9 and FLEX Cre-switch strategy for the conditional expression of a mislocalized Tdp-43∆NLS variant of mouse Tdp-43. This variant is expressed either in the whole body (WB) or specifically in the motor neurons (MNs) in two distinct models. These mice exhibit loss of nuclear Tdp-43, with concomitant cytosolic accumulation and aggregation in targeted cells, leading to increased DNA double-strand breaks (DSBs), signs of inflammation, and associated cellular senescence. Notably, unlike WT Tdp-43, which functionally interacts with Xrcc4 and DNA Ligase 4, the key DSB repair proteins in the non-homologous end-joining (NHEJ) pathway, the Tdp-43∆NLS mutant sequesters them into cytosolic aggregates, exacerbating neuronal damage in mouse brain. The mutant mice also exhibit myogenic degeneration in hindlimb soleus muscles and distinct motor deficits, consistent with the characteristics of motor neuron disease (MND). Our findings reveal progressive degenerative mechanisms in motor neurons expressing endogenous Tdp-43∆NLS mutant, independent of Tdp-43 overexpression or other confounding factors. Thus, this unique Tdp-43 KI mouse model, which displays key molecular and phenotypic features of Tdp-43 proteinopathy, offers a significant opportunity to characterize the early-stage progression of MND further and also opens avenues for developing DNA repair-targeted approaches for treating TDP-43 pathology-linked neurodegenerative diseases.

**Supplementary Information:**

The online version contains supplementary material available at 10.1186/s40478-025-01962-9.

## Introduction

Neuronal degeneration in the central nervous system (CNS) linked to TAR DNA-binding protein of 43kD (TDP-43) pathology is a prominent hallmark of several neurodegenerative diseases, including amyotrophic lateral sclerosis (ALS) [1] and frontotemporal degeneration (FTD) [2]. TDP-43 pathology also features in nearly half of Alzheimer’s disease (AD) cases [3–6]. Classic ALS and FTD pathologies are distinguished by TDP-43 inclusions that are ubiquitin-positive but tau-negative [7]. Several dozen missense mutations, including both familial and sporadic, have been identified in TDP-43, primarily in the disordered prion-like domain (PLD) located at the C-terminus [8, 9]. Two consistent phenomena observed in TDP-43 pathology-linked ALS and FTD patients’ CNS tissues are the nuclear clearance and cytosolic buildup of pathogenic TDP-43 forms. The C-terminal domain (CTD) of TDP-43, while not fully characterized, was shown to interact with various cellular protein complexes, including heteronuclear ribonucleoproteins (hnRNPs), involved in a variety of cellular processes [10].

Progressive accumulation of genome damage and neuroinflammation has been consistently observed in various ALS/FTD disease models as well as in patients’ brain and spinal cord specimens [11–17]. Studies, including ours, reveal that TDP-43 plays a crucial role in executing the non-homologous end-joining (NHEJ)-mediated DNA double-strand break (DSB) repair and in resolving R-loops, both of which are implied in ALS/FTD pathology [11, 18–21]. In this context, TDP-43’s bipartite nuclear localization (NLS) and nuclear export (NES) signal sequences in association with other accessory proteins such as importin-α1/β [22], play pivotal roles in maintaining homeostasis of the TDP-43 protein content across various cellular compartments [23, 24]. Notably, the TDP-43 A90V mutation located within the NLS has been identified in familial ALS/FTD [25]. Thus, altering the NLS could recapitulate the pathology of TDP-43’s nuclear clearance, potentially mirroring ALS/FTD phenotypes in animal models.

While various TDP-43 NLS mice models have been developed to date, each has specific technical limitations in closely mirroring the patient pathology. Most of these transgenic models include the cytoplasmic aggregates of TDP-43 for pathological signaling but not the loss of its nuclear function. For instance, the transgenic Tet-OFF-CamkIIa-hTDP-43-ΔNLS model [26] may not reproduce the effects of endogenous Tdp-43 proteinopathy at the molecular level due to variable interaction and RNA processing abilities of human TDP-43-WT or -ΔNLS in the presence of background murine Tdp-43. Moreover, this model lacks the ability to induce TDP-43 proteinopathy in the spinal cord. Another Tet-OFF-NEFH-hTDP-43-ΔNLS model that induces TDP-43 aggregation in both the brain and spinal cord to study the impact on murine Tdp-43 [27] showed similar limitations. Although another Tdp-43 mouse model of ALS shows Tdp-43 mislocalization associated, this model involves Cre-lox-mediated deletion of exons 2 and 3 encoding the RNA-recognition motif (RRM) regions in addition to NLS sequence, resulting in human ALS irrelevant alternative splicing and RNA processing defects [28]. Additionally, other ALS-Tdp-43 models with mutations in the C-terminal prion-like domain of murine Tdp-43 sequence [29, 30], likely involves altered spliceosomal complex formation capacity of endogenous Tdp-43 and associated alternative splicing of target mRNA transcripts rather than Tdp-43 aggregation-induced changes in gene expression patterns. Thus, there is an urgent need for a suitable *Tardbp* mouse model that can delve into the progression of Tdp-43 toxicity in specific developmental stages or tissue/cell types. This is vital for understanding ALS/FTD’s pre- to post-symptomatic transition.

To confront the challenges of studying the age-associated progression of ALS and deciphering the pathological cellular modifications at the onset or pre-symptomatic stages, we developed a new endogenous conditionally expressing Tdp-43∆NLS (∆82–98 aa) mouse model of ALS. This model allows us to initiate Tdp-43∆NLS expression endogenously in any desired cell/tissue type and developmental stage simply by cross-breeding the Tdp-43∆NLS strain with an appropriate Cre-expressing mouse strain. In this study, we demonstrate that this model effectively captures the key pathological hallmarks of ALS, including TDP-43 aggregation, genomic instability, neuroinflammation, senescence, and muscle wasting, while offering a promising approach for exploring the onset of DNA repair impairment and its associated pathology in ALS/FTD. Furthermore, this model provides a foundation for exploring early-stage ALS/FTD treatment strategies.

## Materials and methods

### Cell culture and treatments

Human neuroblastoma SH-SY5Y cells (ATCC, #CRL-2266) were cultured in DMEM/F12 supplemented with 10% fetal bovine serum (Sigma) and 1% penicillin–streptomycin (Gibco) at 37 °C with 5% CO_2_. SH-SY5Ycells were differentiated in 10 μM retinoic acid (RA) and 50 ng/mL BDNF in DMEM/F12 with 1% FBS for 5 days [11]. Doxycycline-inducible TDP-43 WT and NLS mutated (mNLS) SH-SY5Y lines were generated by transfecting pCW-TDP-43 plasmids using Lipofectamine-3000 (Life Technologies) and selecting against puromycin (Invivogen). For TDP-43 expression, Dox was given at 3 µg/mL final concentration for 72 h under the differentiated condition. TDP-43 downregulation was achieved by RNA interference to TDP-43 (siTDP-43) as described elsewhere [11].

### Comet assay

The neutral comet assay was performed using the Comet Assay Kit (Trevigen, #4250–50-K), according to the manufacturer’s protocol to assess the extent of DNA DSBs in each sample. Briefly, the singlet cell suspension was prepared by trypsinization of control and induced SH-SY5Y cells in DPBS buffer and about 200 cells were smeared in LMAgarose at 37 °C in duplicate in each slide. The comet tails were visualized by staining the DNA with SYBR Green gold stain under a fluorescence microscope.

### Immunoblotting (IB)

Cells were harvested, pelleted at 1,500 rpm at 4 °C for 5 min, and lysed with whole cell lysis buffer (50 mM Tris–HCl pH 7.5, 150 mM NaCl, 1 mM EDTA, and cocktail protease inhibitors). For snap-frozen mice tissues, samples were ground using mortar, pestle, and liquid nitrogen. Then approximately 20 mg powdered tissue samples were lysed in 200 µL of 1 × RIPA lysis buffer added with cocktail protease and phosphatase inhibitors (Roche), centrifuged at 14,000 rpm at 4 °C for 15 min 3–4 times until the white fat pellet was completely cleared. Protein concentration was estimated by Bradford assay. About 20 µg of protein solutions were taken from each sample Protein concentration was determined using the Bradford assay (Sigma). Protein bands were separated on a NuPAGE 4–12% Bis–Tris Gel (Invitrogen). Proteins were electro-transferred onto a nitrocellulose membrane in 1 × NuPAGE transfer buffer. For dot blotting, equal amounts of protein from each sample were taken in 10 µL total volume and blotted on the nitrocellulose membrane, followed by air drying and proceeding to the antibody probing steps. As the positive control, 5 µL (1 µg) of anti-Titin antibody was used and for negative control 1 µg of mouse normal IgG was blotted.

After blocking with 5% skimmed milk solution in 1% Tris-Buffered saline with Tween 20 (TBST) buffer, the membranes were immunoblotted with mouse anti-Flag antibody (Sigma, #F3165, 1:1000), rabbit anti-TDP-43 (Proteintech, #10,782–2-AP, 1:1000), mouse anti-phospho-Histone H2AX (S139) (EMD Millipore, #16–193, 1:1000), rabbit anti-H2AX (Cell Signaling Tech, #2595, 1:1000), rabbit anti-phospho-ATM (S1981) (Abcam, #ab81292, 1:800), rabbit anti-ATM (Abcam, #ab32420, 1:1000), rabbit anti-phospho-53BP1 (S1778) (Cell Signaling Tech, #2675, 1:1000), rabbit anti-53BP1 (Cell Signaling Tech, #4937, 1:1000), mouse anti-TDP-43 (R&D systems, #MAB7778, 1:1000), rabbit anti-phospho-TDP-43 (S409/410) (Proteintech, #22,309–1-AP), mouse anti-Titin (Santa Cruz Biotech, #sc-271946, 1:200), rabbit anti-Stathmin 2 (STMN2; Invitrogen, #720,178, 1:500), mouse anti-GAPDH (Novus, #NBP1-47,339, 1:2000), rat anti-Tubulin (Abcam, #ab6160, 1:2000), and mouse anti-β-actin (Proteintech, #66,009–1-Ig, 1:5000) antibodies. Protein bands were visualized by probing with corresponding HRP-conjugated or IRDye secondary antibodies and developed with enhanced chemiluminescence reagent or at appropriate IR channels in Odyssey (LI-COR). Protein bands were analyzed using Image Studio v5.2 software (LI-COR).

### Generation of Tdp-43∆NLS and bigenic Cre::Tdp-43∆NLS mice

The endogenous Tdp-43∆NLS mice were generated by injecting a linearized plasmid carrying murine *Tardbp* NLS-deleted Exon3 (mExon3) in the reverse orientation following the WT Exon3 and flanked by pairs of WT and mutant loxP sequences with a 5’- (intronic region of –2 kb between Exon2 and Exon3) and 3’- (–2 kb region downstream of Exon3) homology arms in the background of C57BL/6. To induce the recombination process, two DNA DSBs were introduced flanking the WT Exon3 by CRISPR/Cas9 technique. The F1 founder line was screened by a standard genotyping PCR using the primer pairs: loxP-Tdp-F 5’-AAAACACTTGCAGAGCAAGCCTGAC-3’ and loxP-Tdp-R 5’-TGGTTGGAGTGATTTTTCTAGTACCCCC-3’ in a touchdown PCR protocol (denaturation at 94 °C for 5 min; 94 °C – 30 s, 67 °C – 30 s, 68 °C – 30 s for 15 cycles; 94 °C – 30 s, 57 °C – 30 s, 68 °C – 30 s for 25 cycles; final elongation at 68 °C – 10 min). The founder line was maintained by backcrossing with non-carrier C57BL/6 mice. Four founder lines were produced. However, only one hemizygous line was used in this study. Other lines were cryopreserved as backup. Next, the bigenic Cre::Tdp-43∆NLS mice were generated by crossing the monogenic Tdp-43∆NLS line with either tamoxifen (TAM)-inducible Ubc-Cre-ERT2 transgenic line (#008085, The Jackson Laboratory) or Mnx1-Cre line (#006600, The Jackson Laboratory) to establish the whole-body (WB) or motor neuron (MN)-specific Tdp-43∆NLS mouse line. The Cre expression under Ubc promoter was induced by intraperitoneally injecting 75 mg/kg of TAM (#T5648; Sigma) or corn oil (vehicle) every other day for 2 consecutive weeks [32].

Genotyping was performed using earpiece DNA as described previously [33]. All mice were housed in ventilated microbarrier cages on racks providing high-efficiency particulate air (HEPA)-filtered air supply to each cage. Animals were kept on a 12-h light–dark cycle with ad libitum access to food and water. All animal husbandry, experiments, and procedures were performed in strict compliance with animal protocols following the NIH Guide for the Care and Use of Experimental Animals and approved by the Institutional Animal Care and Use Committee (IACUC) of the Houston Methodist Research Institute (Protocol # IS00006797) as well as following the current laws for laboratory animal care and handling of the United States.

### Immunohistochemistry (IHC)

Mice brain, spinal cord, and soleus muscle tissues were immediately harvested after anesthesia with 30% Isoflurane, and half of the tissue samples were snap-frozen in liquid nitrogen for genetic and biochemical analysis, while the other half were stored in 4% paraformaldehyde (PFA) in 0.1 M phosphate buffer for IHC and IF analysis. Tissue samples were paraffin-embedded, sliced into 5 μm horizontal sections and mounted on glass slides. Slides were dewaxed and autoclaved for 10 min at 121 °C in 0.01 M citrate buffer pH6.0 for antigen retrieval. Immunostaining was performed using overnight incubation at 4 °C with mouse anti-TDP-43 (R&D Systems, #MAB77781, 1:200), rabbit anti-TDP-43 (Proteintech, #10,782–2-AP, 1:250), rabbit anti-phospho-TDP-43 (S409/410) (Proteintech, #80,007–1-RR, 1:300), anti-phospho-Histone H2.AX (S139) (Abcam, #ab81299, 1:200), mouse anti-Glial Fibrillary Acidic Protein (GFAP) (Proteintech, #60,190–1-Ig, 1:100), rabbit anti-Iba1 (Fujifilm Wako, #019–19741, 1:300), mouse anti-p62 (Biolegend, #814,801, 1:100), mouse anti-DNA Ligase IV (Santa Cruz Biotech, #sc-271299, 1:50), and mouse anti-XRCC4 (Santa Cruz Biotech, #sc-271087, 1:50). The Nissl staining was performed using NeuroTrace 435/455 Blue Fluorescent Nissl Stain (Invitrogen, #N21479, 1:700).

### Immunofluorescence (IF)

Cells were cultured in 8-well chamber slides (Millicell EZ slides, Millipore), fixed in 4% PFA in phosphate-buffered saline (PBS) for 20 min and permeabilized with 0.5% Triton X-100 in PBS for 20 min at room temperature. For tissue sections, slides were first subjected to the antigen retrieval condition, followed by permeabilization for 30 min at room temperature. After that, slides were blocked in a blocking solution containing 1% gelatin (Sigma-Aldrich, #G7041), 10% normal serum, and mouse IgG (1:500) in 1 × TBS-T solution for 1 h at room temperature under gentle shaking conditions. Primary antibody incubation was carried out using 1% gelatin and 2% serum in 1 × TBS-T overnight at 4 °C and fluorescent secondary antibodies for 1 h at 37 °C. Goat anti-mouse or rabbit Alexa Fluor 488 or 680 conjugated secondary antibodies were used as secondaries (1:500, Molecular Probes, Invitrogen). Slides were washed thrice and counterstained with DAPI. Nuclei were counterstained with SlowFade™ Diamond Antifade Mounting media with DAPI (Invitrogen, #S36964). Images were captured either in a confocal laser scanning microscope (FluoView 3000; Olympus) or Zeiss AXIO Observer and analyzed using the analytical software tools as well as ImageJ (NIH), wherever applicable.

### Proximity ligation assay (PLA)

Paraffin-embedded mouse brain and spinal cord tissue samples were de-paraffinized, antigen retrieved, and permeabilized in permeabilization buffer containing 1% Triton X-100 and 1% gelatin in 1 × TBS-T buffer for 30 min at room temperature. In situ protein–protein interaction was analyzed using a PLA (Duolink) kit, as per the manufacturer’s instructions [11]. Images were analyzed in an AXIO Observer inverted microscope (Carl Zeiss).

### Thioflavin-S staining

Each tissue section was incubated in 500 µM of thioflavin-S (Sigma-Aldrich, #T1892) solution, dissolved in 50% ethanol, for 7 min at room temperature, as described elsewhere [34]. Hoechst-33342 (10 µg/mL, Sigma-Aldrich) was used to observe the nuclear morphology. Images were captured and analyzed using the AXIO Observer inverted microscope. The number and area of plaques detected by thioflavin-S were quantified using ImageJ software.

#### Histopathology

Following the manufacturer’s protocol, mouse brain, spinal cord, and soleus muscle tissue sections were stained with hematoxylin and Eosin (H&E; Abcam, #ab245880) and Congo Red (Abcam, #ab150663).

#### Senescence assay

The CellEvent™ Senescence Green Detection Kit (Invitrogen, C10850) was optimized at 1:800 for 3 h at 37 °C for mouse brain and hindlimb soleus muscle tissue sections of 5 µm thickness. Neurons were identified by co-staining the slides with NeuroTrace Nissl staining reagent.

#### TUNEL assay

The Click-iT™ Plus TUNEL Assay Kit (Invitrogen, #C10617) was used for in situ detection of DNA DSBs in the nuclear genome of cells from brain and spinal cord sections, according to the manufacturer’s instructions [11]. TUNEL images were taken under a brightfield microscope and analyzed using ImageJ software.

#### Long amplicon PCR (LA-PCR) assay

Genomic DNA was isolated from sham and ALS mouse brains using DNeasy Blood and tissue kit (Qiagen) per the manufacturer´s directions. DNA was quantified using Quant-iT™ PicoGreen™ dsDNA Kit (Invitrogen, #P7589) [11]. The accumulation of DNA strand breaks was measured by PCR amplification of long amplicons using LongAmp Taq DNA polymerase (New England Biolabs, #M0323) and three pairs of primers amplifying distinct genomic regions of *polβ*, *Neurod1*, and *Nanog* genes, as described elsewhere [11, 35]. For the LA-PCR assay, 20 mg of tissue powder was used for each sample to extract high-quality genomic DNA using a genomic-tip 20/G kit per the manufacturer’s directions. The thermal cycling profile and DNA concentrations were optimized before setting up the actual reaction. 20 ng of genomic DNA were used for each sample for LA-PCR assay using the optimized thermal profile 94 °C for 30 s (94 °C for 30 s, 59 °C for 30 s, 65 °C for 10 min) for 24 cycles, and 65 °C for 10 min. Internal primer pair (forward: 5´-TATGGACCCCCATGAGGAACA-3´; reverse: 5´-AACCGTCGGCTAAAGACGTG-3´) was used to normalize template DNA across the samples. PCR products were separated in agarose gel and visualized using the Gel Doc XR + (BIO-RAD) system. DNA amplicon bands were quantitated by dsDNA PicoGreen™ assay, as mentioned earlier.

#### Quantitative real-time PCR (qRT-PCR) assay

RNA was extracted from cortical tissue samples using the RNeasy kit (Qiagen), and cDNA was reverse transcribed using oligo(dT) primers and Superscript III (Invitrogen). qRT-PCR was performed using 5 µM of each primer with Power SYBR Green master mix on an ABI Prism 7700 real-time PCR machine (Applied Biosystems). Expression levels of murine endothelin 1 (Edn1), p21, ankyrin 1 (Ankrd1), interleukin-6 (Il-6), and tumor necrosis factor α (Tnf-α) were measured using respective primer pairs, as described elsewhere [36] or designed as follows:

mAnkrd1-F: 5’-AGACTCCTTCAGCCAACATGATG-3’.

mAnkrd1-R: 5’-CTCTCCATCTCTGAAATCCTCAGG-3’.

mEDN1-F: 5’-GCACCGGAGCTGAGAATGG-3’.

mEDN1-R: 5’-GTGGCAGAAGTAGACACACTC-3’.

and normalized to the internal control murine Gapdh level. Relative expression levels were expressed using the 2^− ∆∆Ct^ method.

#### Rotarod

Mice were trained for 3 days and tested following the procedures described elsewhere [37]. Briefly, the mice were placed on a rod (Ugo Basile Rota-Rod 47,600) rotating at a constant speed of 8 rpm. In the testing phase, the rotation speed was accelerated from 8 to 30 rpm in 5 min. When the mice fell from the rod, the latency and fall-off rpm of each mouse were recorded.

#### Hindlimb-clasping test

The mice were suspended by grasping their tails, and their hindlimb positions were observed for 20 s, as described previously [38]. The normal mice consistently kept their hindlimbs away from the abdomen. During the suspended time, the hindlimbs of the MN-Tdp-43∆NLS mice exhibited abnormal movement in their left or right hindlimbs.

#### DigiGait treadmill test

Locomotor performance was assessed using the DigiGait motorized transparent treadmill (Mouse Specifics, Inc.), which allows the recording of animals from both a ventral and lateral view. Mice were pre-trained on the treadmill at speeds of 20 cm/sec and then tested weekly for analysis of the diseased state. Age-matching Ubc-Cre::Tdp-43∆NLS mice were tested before and after the administration of TAM, whereas MN-specific Tdp-43∆NLS mice were evaluated for their motor functions at 6- and 12 months of age. After a 2-min acclimatization to the treadmill, mice were recorded at a speed of 22 cm/sec for 10–15 s in three consecutive trials, with 2-min rest periods between recordings.

#### Statistical analysis

All statistical analyses were carried out using the GraphPad Prism 10.0 software. Data are expressed as mean ± standard deviation (SD) or standard error mean [39], as appropriate. The statistical significance of the results was determined by a one-way or two-way ANOVA, multiple paired t-tests, or Welch’s t-test, as appropriate. A P-value of less than 0.05 was considered statistically significant.

## Results

### Loss of nuclear TDP-43 affects genomic stability in neuronal cells

**Fig. 1:**
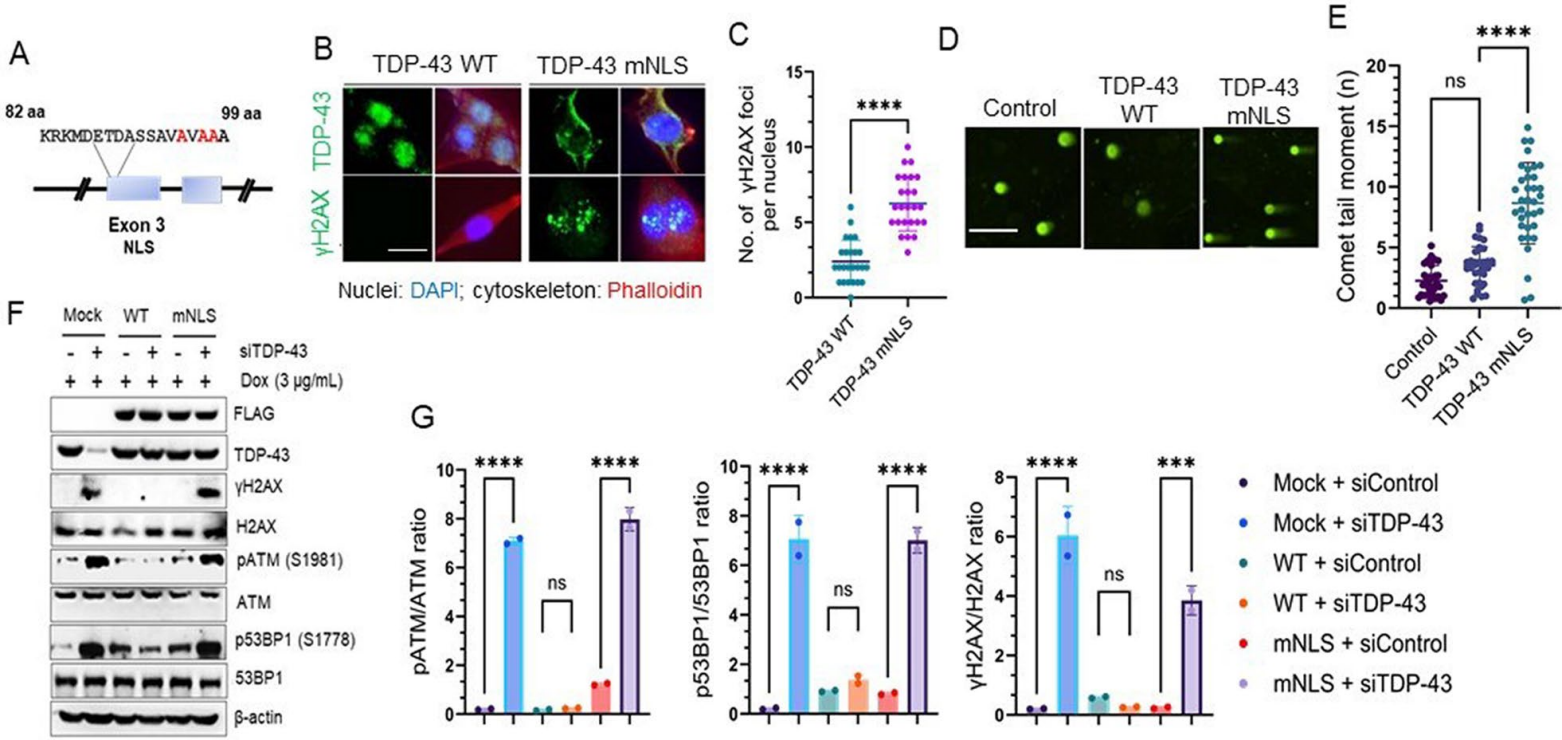
Inactivation of the TDP-43 nuclear localization signal (NLS) induces genomic instability. (**A**) Schematic presentation of NLS point mutations (K95A, K97A, and R98A) in TDP-43. (**B-C**) Immunofluorescence (IF) analysis of TDP-43 localization and DNA double-strand breaks (DSB) in doxycycline-inducible TDP-43 wild-type (WT) and NLS-inactivated (mNLS) neuronal cells using anti-TDP-43 (*upper panel*) and anti-γH2AX (S139) antibodies (*lower panel*). Nuclei were stained with DAPI and cytoskeleton with Alexa-Flour 568 Phalloidin. Scale bar = 10 µm (**B**). Quantitative analysis of γH2AX foci counts in the nucleus (n = 25 cells in each experiment) (**C**). Data were analyzed using a t-test from two independent experiments (N = 2); mean ± SEM, ****, *P* < 0.0001. (**D-E**), Neutral comet assay showing TDP-43 mNLS expression-associated DSB accumulation in neuronal cells compared to TDP-43 WT cells. Scale bar = 20 µm (**D**). Quantitation of comet tail moments for each experimental group (n = 50 cells); one-way ANOVA; ****, *P* < 0.0001 (**E**). (**F-G**) Doxycycline (Dox)-induced WT or mNLS TDP-43 expressing neuronal cells were transfected with antisense RNA (siRNA) to the 3’UTR of *TARDBP* mRNA (siTDP-43) or control siRNA (siControl) and harvested at 72 h post-transfection for immunoblotting (IB) analysis using indicated antibodies (**F**). Quantitation of phosphorylated to total protein expression ratio from two independent experiments (N = 2) by one-way ANOVA (**G**). ns, non-significant; ***, *P* < 0.001; ****, *P* < 0.0001

Mimicking TDP-43 neuropathology in experimental models poses a dual challenge: the loss of nuclear TDP-43 indicating a loss-of-function (LOF) phenotype coupled with its accumulation in the cytosol, inducing a gain-of-toxicity [40] in impacted neurons [2, 41]. To attribute our previously reported DNA damage phenotype to the loss of nuclear TDP-43 [11], we generated a doxycycline-inducible SH-SY5Y cell model ectopically expressing either WT or NLS mutant human TDP-43 (hereafter referred to as hTDP-43 mNLS) (Fig. 1A). Dox induction (3 µg/mL) for 72 h showed robust nuclear clearance of flag-tagged hTDP-43 mNLS (Fig. 1B, *upper panel*) together with a significant increase (3.840 ± 0.4593; *P* < 0.0001) in γH2AX foci formation (Fig. 1B-C) compared to the hTDP-43 WT form. We further validated a higher baseline of genome damage in the mNLS line, using neutral comet assay to estimate endogenous DNA DSBs in these cells (Fig. 1D-E). Analysis of comet tail moments consistently revealed –2.47 fold (*P* < 0.0001) higher population of mNLS cells with unrepaired DSBs even in the absence of any exogenous genome damaging agents (Fig. 1E). Next, we explored the status of the DNA damage response (DDR) pathway in the WT versus mNLS lines without any exogenous damage. Being an auto-regulatory protein, ectopically expressed TDP-43 is expected to maintain homeostasis of endogenous TDP-43 level [42]. Thus, we depleted the endogenous TDP-43 level by a small interfering RNA against *TARDBP* (siTDP-43) to evaluate the genome-damaging effect of the mNLS variant solely in comparison to the WT protein. Immunoblotting (IB) results showed that levels of at least three classical DDR markers – phospho-ATM (S1981), and phospho-53BP1 (S1778), and γH2AX (S139) – were significantly upregulated in siTDP-43-treated mock and mNLS cells but not in TDP-43 WT cells (Fig. 1F-G). Because overexpression of TDP-43 WT is toxic to cells [26, 43, 44], by downregulating the background TDP-43 level, we confirmed that the observed effects were primarily due to the expression of the mNLS variant but not to its overexpression level. These findings, together with our previous reports, suggest that both abnormal cytosolic accumulation as well as nuclear loss of functional TDP-43 collectively contribute to the DNA damage/repair imbalance, as observed in ALS/FTD pathology, which prompted us to develop a conditional cytosolic mislocalization murine Tdp-43 model of ALS by manipulating the murine *Tardbp* gene to better unravel the pathogenic mechanism of human ALS/FTD.

### Development of endogenous Tdp-43ΔNLS knock-in (KI) mouse model

**Fig. 2:**
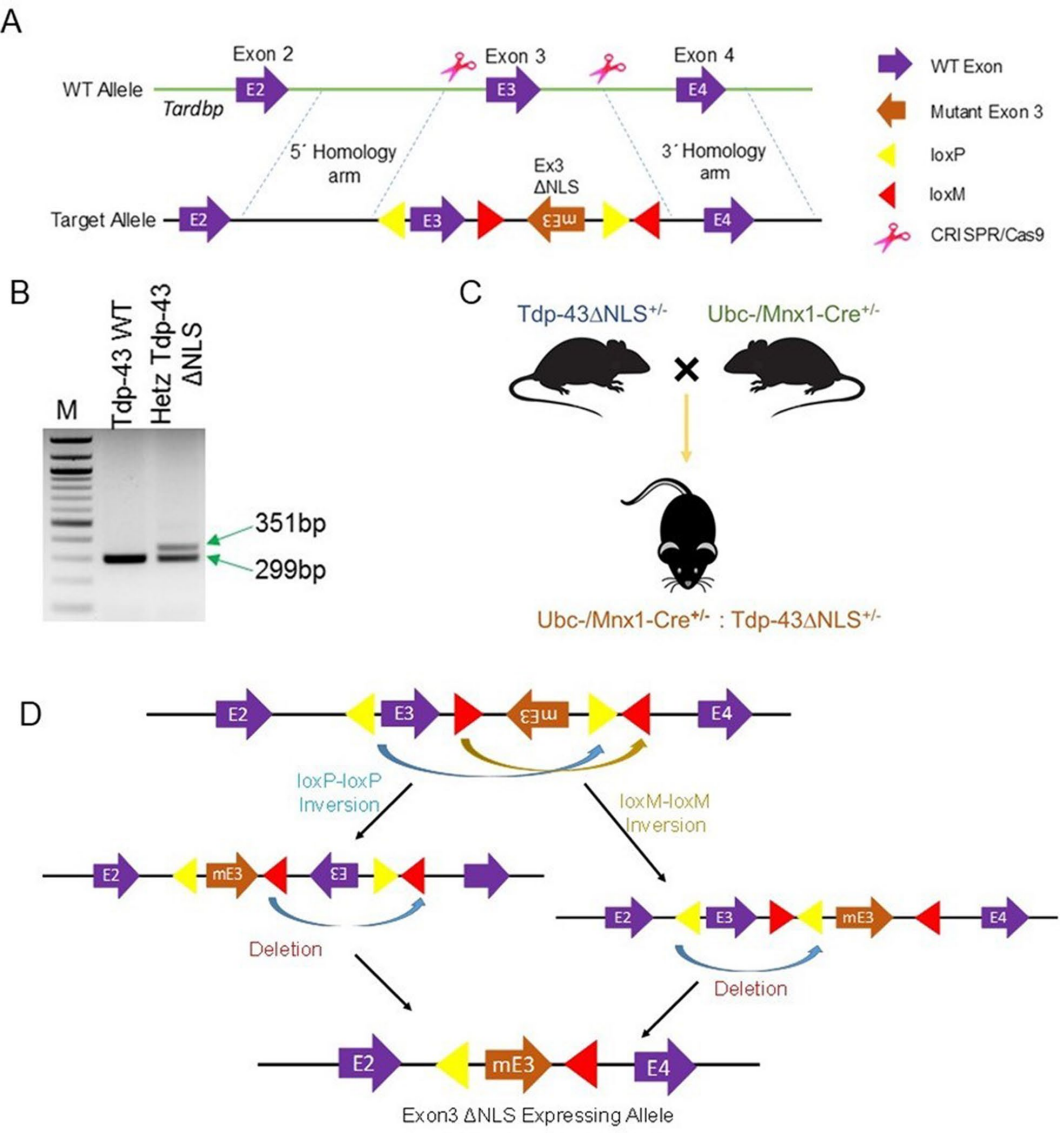
Generation of Tdp-43 knock-in mouse model. (**A**) Illustration of the target allele design for the murine *Tardbp* gene. (**B**) Genotyping PCR identifies a wild-type and a heterozygous littermate where the band size of 351 bp indicates the presence of the floxed target allele. (**C**) Schematic of the double-heterozygous Cre^**±**^:Tdp-43∆NLS^±^ strain generation. (**D**) Illustration of the Cre-mediated recombination of floxed WT and mutant Exon-3 deletion and re-orientation, respectively, resulting in the expression of mutant Tdp-43∆NLS variant in the desired cell type in the central nervous system (CNS)

In our continued efforts to mirror the mislocalization and collective effects of LOF and GOT of TDP-43, especially under non-overexpressing conditions, we embarked on the creation of a unique conditional CRISPR/Cas9-mediated KI mouse model expressing Tdp-43∆NLS variant at the endogenous level. In this cis-genic Tdp-43ΔNLS mouse model, the NLS-lacking mEx-3 flanking a converging pair of lox2272 (loxM) sequences was placed in reverse orientation (3ʹ → 5ʹ). In contrast, the normal Ex-3 was flanked by a diverging pair of WT loxP sequences (Fig. 2A). We confirmed this genetic modification through genotyping with a pair of primers amplifying the 5’ loxP sequence on the target allele (Fig. 2B). This model was designed to conditionally trigger the replacement of murine *Tardbp* Exon-3 with NLS-deleted mutant Exon-3 (mEx-3) through the FLEX Cre-switch strategy (Fig. 2C-D). Thus, we established the Tdp-43∆NLS model of ALS by crossing heterozygous Tdp-43∆NLS^±^ mice strain with TAM-inducible Ubc (whole body, WB) or Mnx1 (motor neuron, MN)-specific promoter-driven Cre^±^ mice as shown in schematics (Fig. 2C). Thereby, we were able to generate two different types of murine Tdp-43∆NLS mice models of ALS: one with inducible Cre-mediated WB-Tdp-43∆NLS expression and the other with developmentally regulated Mnx1-driven Cre expression-mediated MN-Tdp-43∆NLS in post-mitotic neurons in the CNS. Given that homozygous Tdp-43 mutation renders the embryo non-viable [45], we used double-heterozygous animals for all experiments related to this study.

### Tdp-43∆NLS expression induces Tdp-43 mislocalization and aggregation pathologies in the CNS

**Fig. 3:**
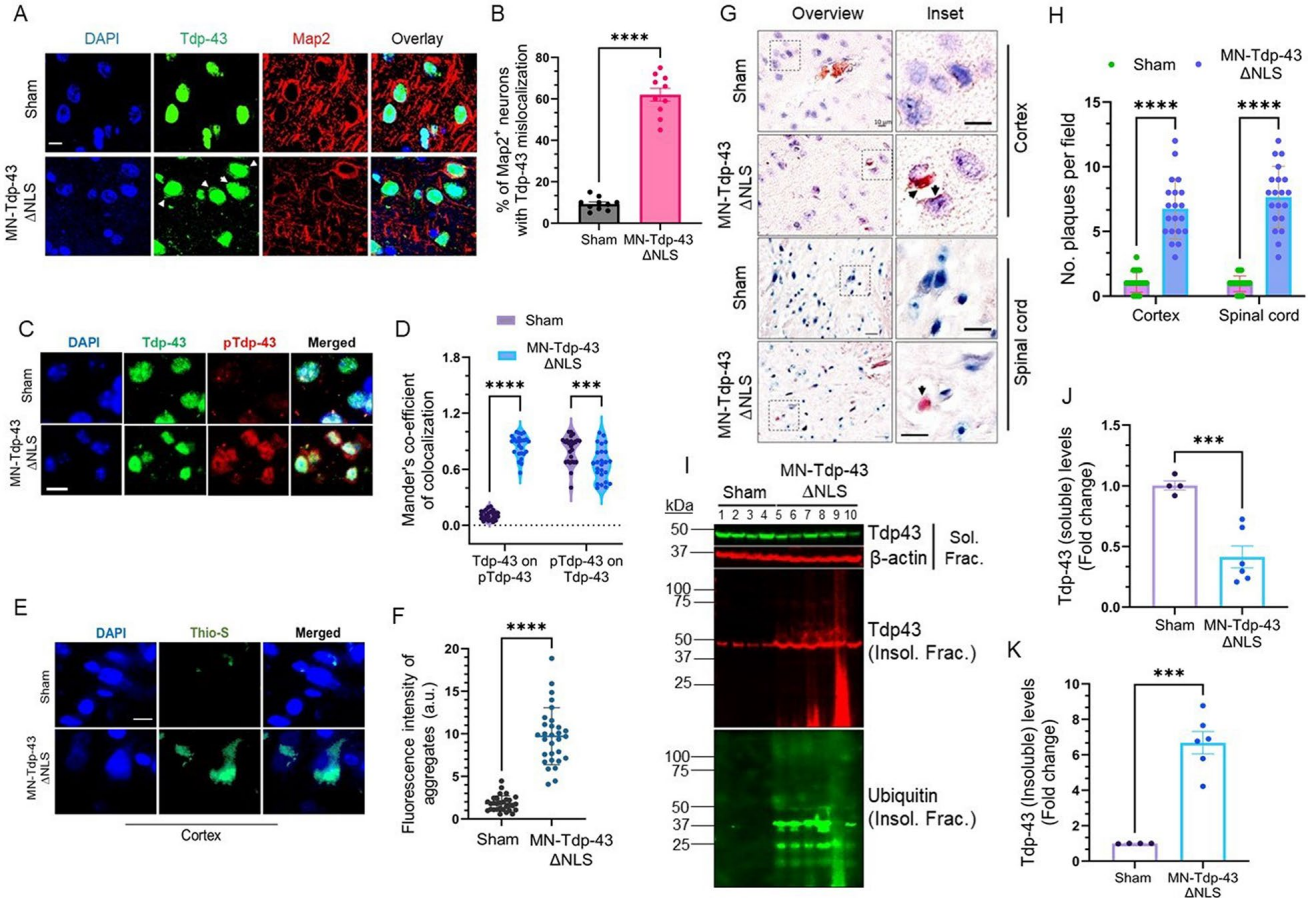
Neuronal Tdp-43∆NLS expression induces Tdp-43 mislocalization and formation of pathological aggregates in the cytosol. (**A-B**) Immunohistochemistry (IHC)-immunofluorescence (IF) staining with anti-Tdp-43 (Alexa Fluor 488) and anti-Map2 (Alexa Fluor 647) antibodies in the cortical brain regions of MN-Tdp-43∆NLS and sham mice (N = 6 mice/group). Nuclei were counterstained with DAPI. Scale bar = 10 µm (**A**). (**B**) Quantitation of percentages of Map2^+^ motor neurons with Tdp-43 mislocalization in the cortex by student’s t-test. ****,* P* < 0.0001. (**C**) Representative colocalization IF images stained with anti-Tdp-43 and anti-phosphoTdp-43 (S409/410) antibodies in cortices of sham and MN-Tdp-43∆NLS mice brains. Nuclei were counterstained with DAPI. Scale bar = 10 µm; N = 25 microscopic fields from 6 mice/group. (**D**) Quantitation of Mander’s coefficient of colocalization of Tdp-43 (Green) on pTdp-43 (Red) signals and vice versa using two-way ANOVA. ***,* P* < 0.001; ****,* P* < 0.0001. (**E–F**) Thioflavin-S staining images of the cortical tissue from sham and MN-Tdp-43∆NLS mice brains. Nuclei were counterstained with DAPI. Scale bar = 10 µm (**E**). (**F**) Quantitation of fluorescence intensity (arbitrary unit, a.u.) of Thioflavin-S-positive aggregates (N = 30 cells; 6 mice/group) by t-test. ****,* P* < 0.0001. (**G-H**) Representative Congo red staining images of the cortex from sham and MN-Tdp-43∆NLS mice. Pink stain indicates amyloid plaques in the cytosol and inter-cellular spaces. Nuclei were counterstained with hematoxylin. Scale bars = 10 µm (overview—cortex), 20 µm (overview – spinal cord), and 10 µm (inset) (**G**). (**H**) Quantitation of the number of amyloid plaques per field. N = 20 different microscopic fields from 6 mice/group by two-way ANOVA. ****,* P* < 0.0001. (**I-K**) IB of sham and MN-Tdp-43∆NLS mice cortical brain lysates to assess levels of monomeric TDP-43 (soluble fraction – Green) and aggregated TDP-43 (insoluble fraction – Red). β-Actin served as the loading control for soluble fractions. The ubiquitination status of proteins was also measured in insoluble fractionates from sham and MN-Tdp-43∆NLS mice by probing with anti-ubiquitin antibody (**I**). (**J-K**) Quantitation of IB band intensities by t-test with Welch’s correction. ***, *P* < 0.001. Data are expressed as mean ± standard deviation (SD)

We examined the neuronal populations displaying murine Tdp-43 mislocalization in the cortex of WB- and MN-Tdp-43∆NLS mice. IHC analysis revealed that 26.6% of brain cells had notable Tdp-43 mislocalization in WB-Tdp-43∆NLS mice, and about 21.3% of the cell population with Tdp-43 mislocalization in MN-Tdp-43∆NLS mice than their respective sham controls (Supplemental Fig. 1A-B; all *P* < 0.0001). To further validate the MN-Tdp-43∆NLS mouse model of ALS, we performed IHC-IF assays by co-staining with anti-Map2 and Tdp-43 antibodies in the deep cortical layers (III-IV), revealing that 53.5% (*P* < 0.0001) more Map2-positive neurons predominantly had the cytosolic Tdp-43 mislocalization phenotype in MN-Tdp-43∆NLS mice than sham mice (Fig. 3A-B), along with distorted MN nuclei and reduced expression of Map2 (-1308 ± 113.2 a.u.; *P* < 0.0001; Supplemental Fig. 1C), suggesting an MND pathology in the brain of MN-Tdp-43∆NLS mice.

Given that TDP-43 proteinopathy primarily affects the MN in ALS and FTD brains, we exploited the MN-Tdp-43∆NLS mouse line for histopathological and biochemical analyses relevant to ALS/FTD-TDP-43 pathology in human patients, unless stated otherwise in the following results. We evaluated the extent of Tdp-43 protein aggregation in the realm of protein mislocalization in the CNS of mutant mice. Unlike in sham mice, the IF co-staining with anti-Tdp-43 and pTDP-43(S409/410) antibodies exhibited a significant overlap of the cytosolic total Tdp-43 and pTdp-43 in the cortex of MN-Tdp-43∆NLS compared to that in sham control as measured by the Mander’s coefficient of colocalization [Tdp-43 on pTdp-43 vs. pTdp-43 on Tdp-43: 0.7411 ± 0.02500 (*P* < 0.0001) vs. -0.1489 ± 0.04628 (*P* < 0.001)] (Fig. 3C-D). Likewise, thioflavin-S staining indicated significantly enhanced protein aggregation in the cytosol compared to that in age-matched sham mice brains (Fig. 3E-F). Furthermore, we examined the status of amyloidogenic plaque formations in the CNS of MN-Tdp-43∆NLS versus sham mice using Congo red staining which exhibited the formation of amyloidogenic plaques in the nuclear periphery and/or cytosol in mutant mice cortices and spinal cord regions compared to that in sham mice (Fig. 3G). In either of the CNS regions, MN-Tdp-43∆NLS mice had significantly higher counts of amyloid plaques per field (cortex: 6.14-fold; spinal cord: 8.05-fold; all *P* < 0.0001) than in age-matched sham (Fig. 3H). Additionally, IB analysis of soluble vs. insoluble fractionates of the cortical samples showed a 2.43-fold (*P* = 0.0007) decrease in the level of soluble Tdp-43 (Fig. 3J) and 6.71-fold (*P* = 0.0003) increase in the level of insoluble Tdp-43 in MN-Tdp-43∆NLS mice compared to sham mice (Fig. 3K). Notably, this strain also exhibited increased levels of ubiquitinated proteins in the insoluble fraction of brain tissues (Fig. 3I-K).

### Tdp-43 mislocalization induces muscle atrophy and gait deformities

**Fig. 4:**
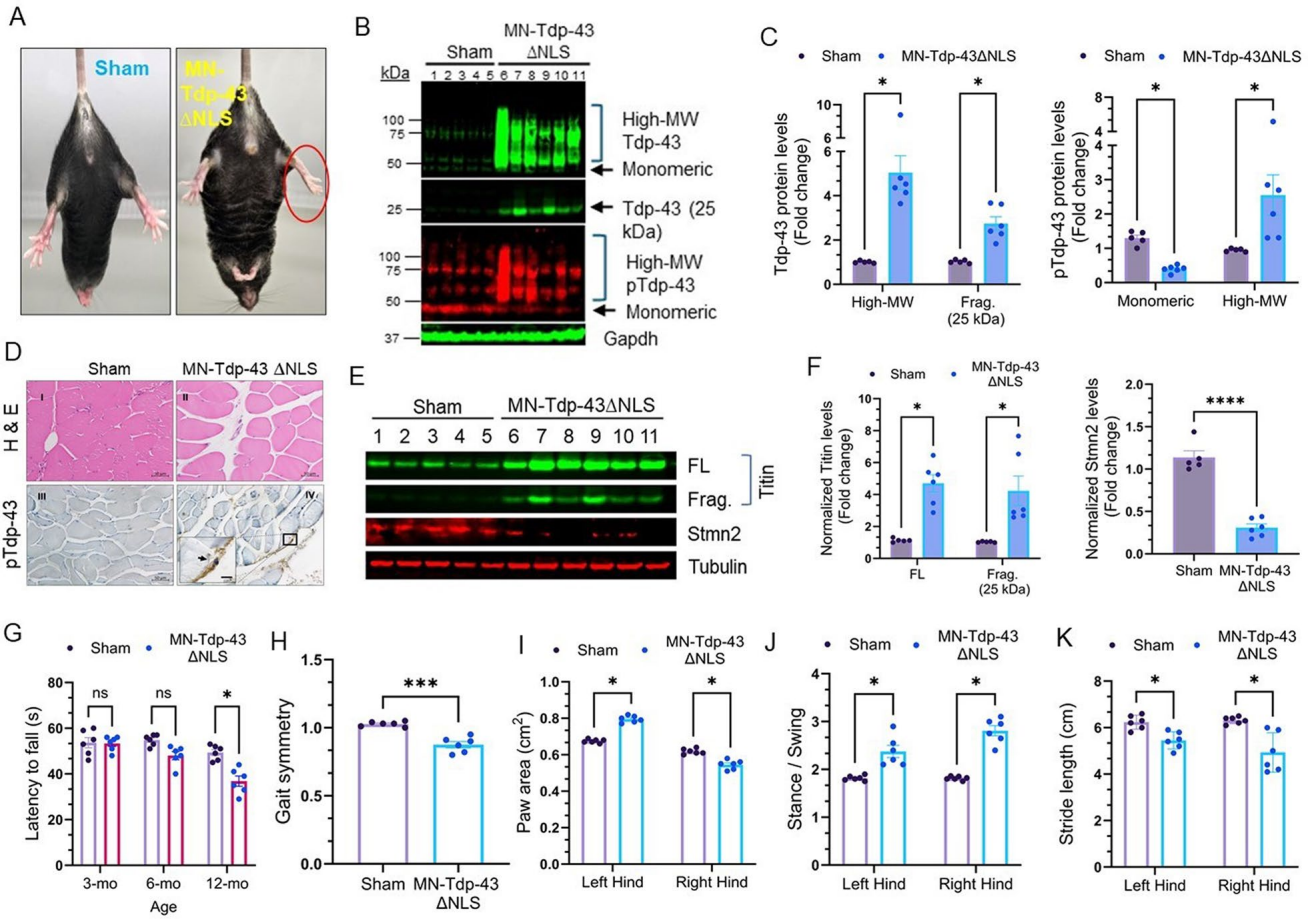
MN-Tdp-43∆NLS expression causes muscle atrophy and gait deficits in Tdp-43 mutant mice. (**A**) Representative live-mice images showing abnormal hindlimb reflexes in 12-month-old MN-Tdp-43ΔNLS mice (N = 6) but not sham mice (N = 5). (**B-C**) IB images exhibiting levels of high-molecular weight (MW) Tdp-43 and pTdp-43 (S409/410), and pathological 25 kDa fragment of Tdp-43. Gapdh served as the loading control (**B**). (**C**) Quantitation of normalized protein levels in fold changes by multiple paired t-tests. *, *P* < 0.05. (**D**) Hematoxylin–Eosin (H&E) staining of sham and MN-Tdp-43ΔNLS mice soleus (I-II) tissues. IHC staining with anti-phosphorylated Tdp-43 (S409/410) antibody in soleus muscle tissues (III-IV). Scale bar = 50 µm. The inset image displays cytosolic pTdp-43 staining in muscle cells. (**E–F**) IB analysis of Titin [full-length: FL; Fragment of 25 kDa: Frag. (25 kDa)] and Stmn2 levels in soleus muscle samples from sham (N = 5) and MN-Tdp-43ΔNLS (N = 6) mice. α-Tubulin served as the loading control (**E**). (**F**) Comparisons of normalized Titin (FL & Frag.) and Stmn2 levels between the two groups using multiple paired t-tests or Welch’s t-test. Data are expressed as mean ± SD. **, *P* < 0.01; ***, *P* < 0.001; ****, *P* < 0.0001. (**G**) Rotarod testing to assess the latency to fall (seconds) for MN-Tdp-43ΔNLS and sham mice (N = 6 mice/group) analyzed by multiple paired t-tests. ns, non-significant; *, *P* < 0.05. DigiGait analyses of MN-Tdp-43ΔNLS versus sham mice (**H**) gait symmetry; (**I**) hindlimb paw area (cm^2^); (**J**) stance-to-swing ratio; and (**K**) stride length (cm). N = 6 mice/group. Data are expressed as mean ± SEM and analyzed by multiple paired t-tests. *, *P* < 0.05; ***, *P* < 0.001

To assess the impact of Tdp-43 proteinopathy on motor functions, we initially conducted a tail suspension test to observe signs of limb clasping behavior in mutant and sham mice. Each mouse was suspended for 30 s, and the test was repeated thrice. Unlike their sham counterparts, MN-Tdp-43∆NLS mice exhibited abnormal hindlimb response to stress (Fig. 4A). These mice did not show any significant differences in their bodyweights compared to the age-matched sham mice, however, at 6 and 12 months of age, we observed increasing variations in their bodyweights, possibly due to Tdp-43 pathology-associated metabolic dysregulations (Supplemental Fig. 2) [46, 47]. There was no significant difference in survival rates of MN-Tdp-43∆NLS and sham mice up to 12 months of age (data not shown). IB analyses of hindlimb muscle tissues (Fig. 4B) indicated significantly increased levels of high molecular weight (MW) forms of total Tdp-43 (4.99-fold; *P* = 0.007888), pTdp-43 (2.96-fold; *P* = 0.049181), and pathological 25 kDa fragment (2.66-fold; *P* = 0.009748) of Tdp-43 in soluble fractionates with subsequent reductions in monomeric pTdp-43 levels (3.22-fold; *P* = 0.001971) in MN-Tdp-43∆NLS mice compared to those in age-matched sham mice (Fig. 4C). Histopathological analysis of tissue sections also revealed disintegrated muscle fiber discs and abnormal distribution of satellite cells due to denervation of motor neurons (Fig. 4D, *upper panel*) and enhanced signal intensity of anti-pTdp-43 antibody staining (Fig. 4D, *lower panel*) in 12-month-old MN-Tdp-43∆NLS mice tissues compared to age-matched sham mice samples. To investigate the molecular phenotype of Tdp-43 pathology in these muscle tissues further, we performed IB analysis to examine the levels of muscle fiber protein Titin and neuro-muscular junction (NMJ)-related protein Stmn2. Interestingly, we noticed significantly increased levels of full-length (4.46-fold; *P* = 0.002764) and 25 kDa fragmented (4.41-fold; *P* = 0.029098) Titin proteins, along with reduced levels (3.69-fold; *P* < 0.0001) of Stmn2 in MN-Tdp-43∆NLS mice than in age-matched sham mice (Fig. 4E-F; Supplemental Fig. 3), which were consistent with previous findings [48–51].

Subsequent evaluation of gait parameters revealed significant differences between the MN-Tdp-43∆NLS and sham groups. First, we measured the latency to fall (seconds) on a rotarod instrument at different age groups – 3-month, 6-month and 12-month, which showed a significant difference (12.50 ± 2.217 s, *P* = 0.007289) in latency to fall [52] only between the two groups at 12-month age (Fig. 4G). MN-Tdp-43∆NLS mice displayed abnormal gait symmetry at a statistically significant level (-0.1517 ± 0.02442; *P* = 0.0008) than their control counterparts (Fig. 4H). Furthermore, DigiGait analysis indicated a significant decrease in the paw area (cm^2^) of the right hind limbs (0.07333 ± 0.01430; *P* = 0.003680) of MN-Tdp-43∆NLS mice relative to sham, with a reversed pattern observed in the left hind limbs (-0.1200 ± 0.006325 cm^2^; *P* = 0.000015) (Fig. 4I). The stance-to-swing ratio was also notably higher in the hind limbs (left: -0.5583 ± 0.1186, *P* = 0.005298; right: -0.9950 ± 0.1073, *P* = 0.000490) of MN-Tdp-43∆NLS mice compared to sham controls (Fig. 4J). While stride length measurement indicated significantly reduced lengths (left: 0.7833 ± 0.1956 cm, *P* = 0.020463; right: 1.383 ± 0.3637 cm, *P* = 0.020463) in MN-Tdp-43∆NLS mice than in sham controls (Fig. 4K). Notably, when we analyzed the paw angle (degrees)and percent (%) swing/stride, we found left hind limb-centered gait defects in WB-Tdp-43∆NLS expressing mice (Supplemental Fig. 4A-C), while abnormal brake (seconds) activities were significantly higher in the right hind limbs of WB-Tdp-43∆NLS mice than in respective sham mice (Supplemental Fig. 4D).

### Tdp-43 proteinopathy associates with neuronal genome damage in mice brain

**Fig. 5:**
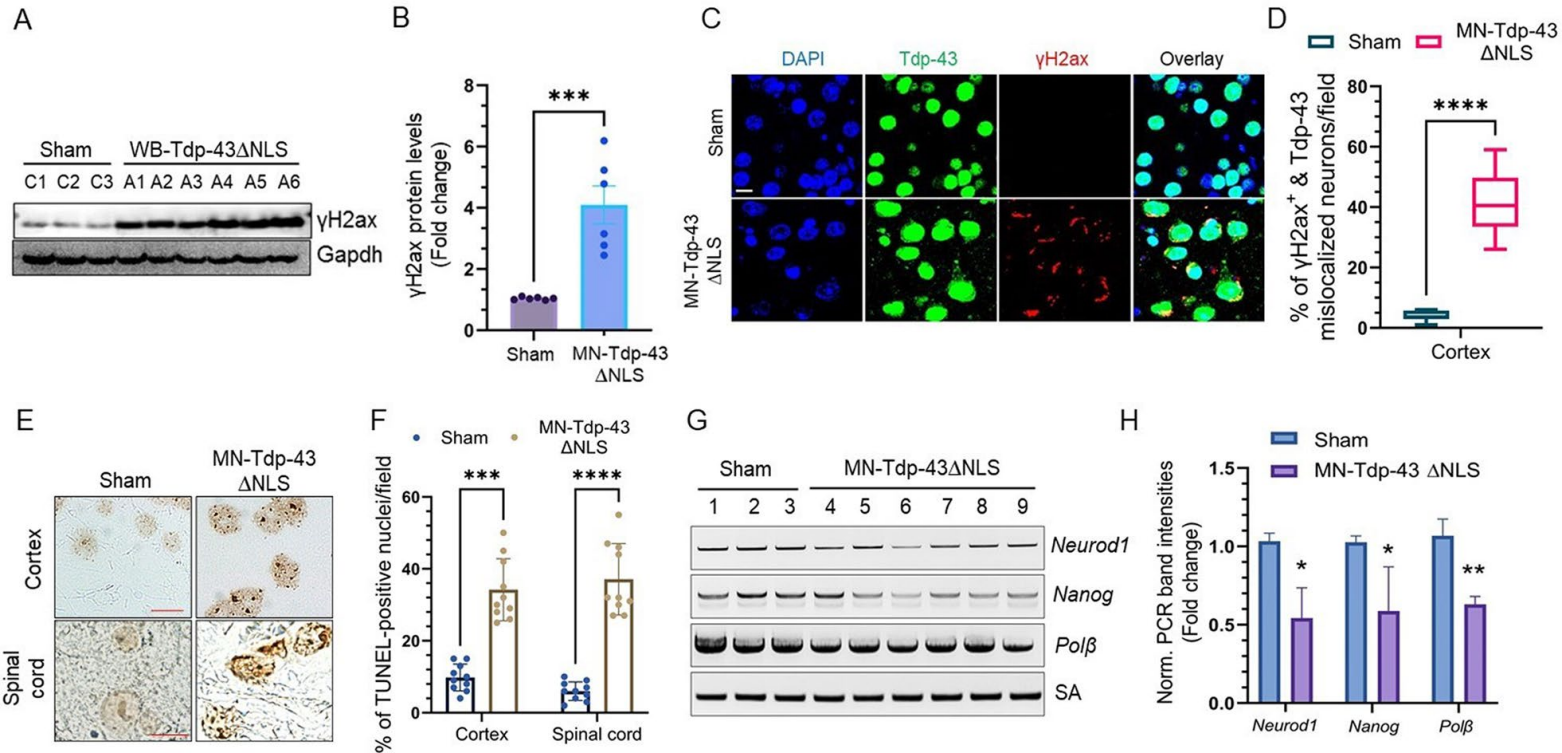
Tdp-43∆NLS induces genome damage in the central nervous system (CNS). (**A-B**) Representative IB images of cortical brain extracts from sham (N = 6) and WB-Tdp-43∆NLS mice (N = 6) using anti-γH2ax. Gapdh served as the loading control (**A**). (**B**) Quantitation of γH2ax protein levels (fold change) of the MN-Tdp-43∆NLS and sham mice groups. (**C-D**) Representative IF images of colocalization of Tdp-43 mislocalization with γH2ax foci using respective antibodies in the cortex of sham and MN-Tdp-43∆NLS mice (N = 6/group). Nuclei were counterstained with DAPI. Scale bar = 10 µm (**C**). (**D**) Quantitation of percent of γH2ax-positive and Tdp-43 mislocalized neurons by t-test. N = 12 different microscopic fields per group at 40 × optical magnification. ****, *P* < 0.0001. (**E–F**) TUNEL analysis to estimate the neuronal genome damage in the cortex and spinal cord of MN-Tdp-43∆NLS expressing mice. Scale bar = 20 µm (**E**)**.** (**F**) Quantitation of the number of cells with TUNEL-positive nuclei by two-way ANOVA. ***, *P* < 0.001; ****, *P* < 0.0001. (**G-H**) Long-amplification PCR amplification (LA-PCR) of –6–8 kb of genomic length from the cortical genome of MN-Tdp-43∆NLS (N = 6) and sham mice (N = 3). A 200 bp short-amplification (SA) product was used as an internal control (**G**). (**H**) Quantitation of normalized PCR band intensities of each genomic target in the MN-Tdp-43∆NLS and sham groups. Data are expressed as mean ± SD and analyzed by multiple t-tests. *, *P* < 0.05, **, *P* < 0.01

Considering the pivotal role of TDP-43 in maintaining genomic integrity and given its mislocalization or nuclear clearance impairs DNA DSB repair in ALS-affected MNs [11], we examined the level of DSB marker γH2ax in cortical tissues of 12-month-old MN-Tdp-43∆NLS and sham mice by IB, which showed about fourfold higher expression of γH2ax in MN-Tdp-43∆NLS mice cortical brain samples than in sham controls (Fig. 5A-B). Furthermore, we sought to recapitulate our *in-cell* results on whether neurons with Tdp-43 mislocalization would predominantly have γH2ax signal in MN-Tdp-43∆NLS mice than their sham counterparts. As expected, IHC-IF co-staining with anti-γH2ax and anti-Tdp-43 antibodies displayed significantly higher percentages (37.50 ± 2.954%; *P* < 0.0001) of neurons with Tdp-43 mislocalization cum γH2ax foci or puncta accumulation in their nuclear periphery in MN-Tdp-43∆NLS mice compared to sham mice, indicating a pro-apoptotic condition in these degenerating neurons (Fig. 5C-D). Additionally, TUNEL analysis also confirmed significantly elevated levels of genome damage as measured by the percent of TUNEL-positive nuclei per microscopic field in the cortex (-24.40 ± 3.541%; *P* = 0.0001) and spinal cord (-31.10 ± 3.261%; *P* < 0.0001) of MN-Tdp-43∆NLS mice than in sham control (Fig. 5E-F). Finally, we performed the long amplification PCR (LA-PCR) analysis to further corroborate that Tdp-43 mislocalization induced accumulation of genome-wide DNA DSBs in neuronal cells by using specific primer pairs for actively transcribing mouse genes *Neurod1*, *Nanog,* and *Polβ*, along with a small amplicon (SA) PCR primer pair as internal control. We found approximately twofold reductions in genome integrity, as indicated by reduced signal intensities of LA products, in cortical samples of MN-Tdp-43∆NLS mice compared to sham controls (Fig. 5G-H).

### Mislocalized Tdp-43 sequesters DNA repair factors in the cytosol of neurons

**Fig. 6:**
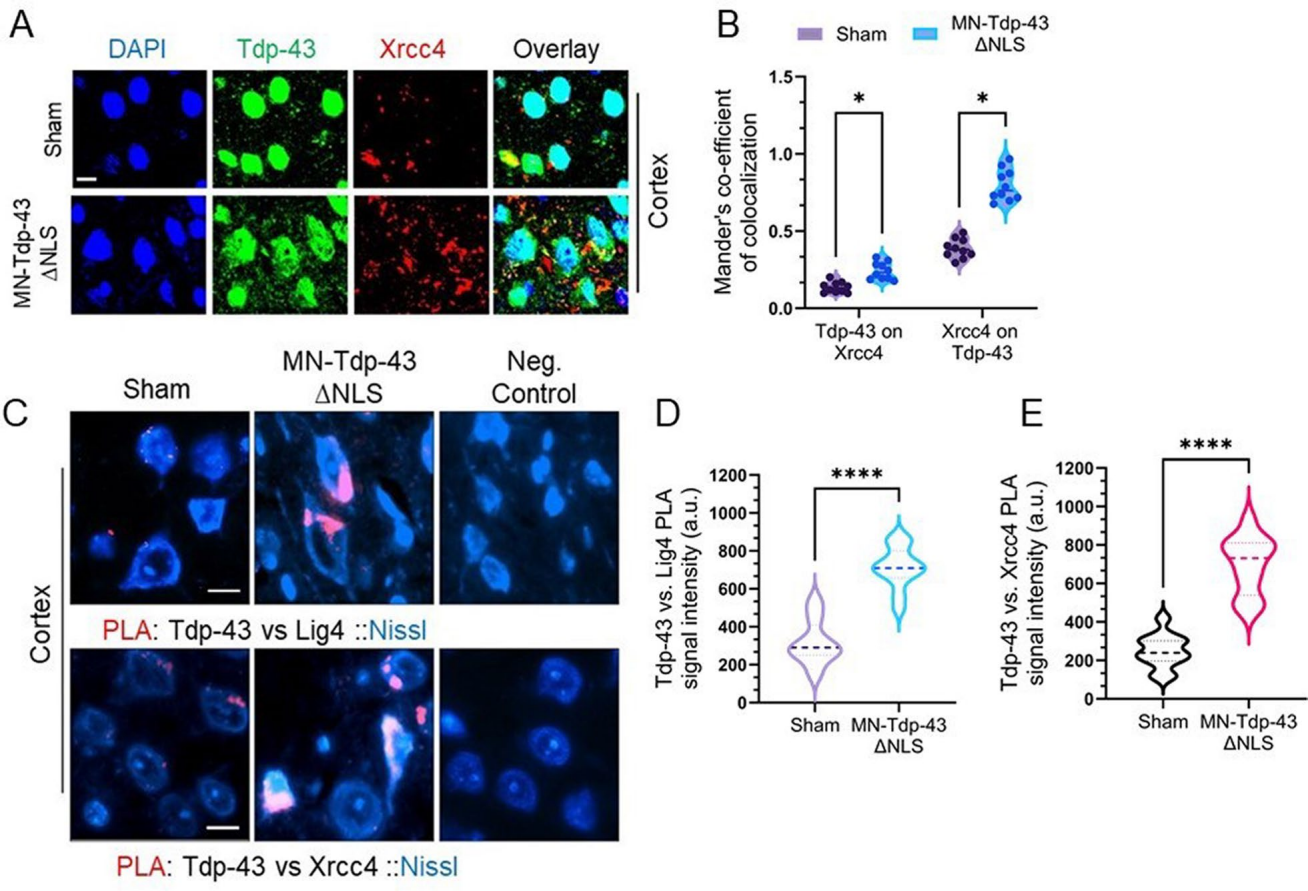
Tdp-43∆NLS causes trapping of Xrcc4 and Ligase 4 in the cytosol of neurons. (**A-B**) Representative IF images of Tdp-43 and Xrcc4 colocalization in the cortex of sham and MN-Tdp-43∆NLS mice (**A**). (**B**) Analysis of the extent of colocalization of Tdp-43 and Xrcc4 IF signals using Mander’s colocalization coefficient. (**C-E**) Representative images of proximity ligation assay (PLA) between Tdp-43 and DNA Ligase 4 (Lig4) or Xrcc4 in the cortex of sham and MN-Tdp-43∆NLS mice. Cell bodies were counterstained with Alexa-Fluor 488-conjugated Nissl stain. PLA signals were visualized as red foci/puncta at 568 nm. Anti-Tdp-43 rabbit antibody was used against mouse normal IgG as negative control. Scale bar = 10 µm. (**C**). Quantitation of PLA signal intensity from 12 different microscopic fields per group for Tdp-43 vs Lig4 (**D**) and Tdp-43 vs Xrcc4 (**E**). Data are expressed as mean ± SEM and analyzed by multiple paired t-tests. *, *P* < 0.05; ****, *P* < 0.0001

We have demonstrated earlier that mislocalized TDP-43 sequesters and prevents the mobilization of the key DNA DSB ligation factors, XRCC4 and DNA Ligase 4 (Lig4), to the nucleus of human cells in response to nuclear genome damage [21]. We sought to explore if similar phenomena could be detected in this novel MN-Tdp-43∆NLS mouse model. To test this hypothesis, we first performed an IHC-IF co-staining with anti-Tdp-43 and anti-Xrcc4 antibodies in the cortical brain sections, and analysis of Mander’s coefficient showed a significantly increased overlap of Tdp-43 and Xrcc4 signal intensities [Tdp-43 on Xrcc4: -0.1066 ± 0.02034, *P* = 0.000535; Xrcc4 on Tdp-43: -0.4090 ± 0.03386, *P* = 0.000001] in MN-Tdp-43∆NLS mice compared to sham controls (Fig. 6A-B). Next, to examine whether these colocalizing proteins are involved in physical interaction, we performed PLA for anti-Tdp-43 versus anti-Lig4, anti-Xrcc4 antibodies or normal mouse IgG and counterstained cell bodies with Nissl stain (Fig. 6C). The results showed that MN-Tdp-43∆NLS mice brain neurons from cortical layers III-IV had significantly strong PLA signal intensities for Tdp-43 vs. Lig4 (395.6 ± 38.94 a.u., *P* < 0.0001) and Tdp-43 vs. Xrcc4 (447.9 ± 53.33 a.u., *P* < 0.0001) compared to respective sham controls (Fig. 6D-E). Furthermore, when we recapitulated the PLA assay between Tdp-43 and Lig4 in spinal cord samples of both strains, puncta were observed in the cytosol of MN-Tdp-43∆NLS mice neurons surrounding the nuclear periphery, unlike the sham mice (Supplemental Fig. 5A-B), suggesting that mislocalized murine Tdp-43 sequesters DNA repair proteins in the cytosol of neurons and causes DNA damage and repair impairment.

### Impaired DNA repair is associated with elevated neuroinflammation and accelerated neuronal senescence in MN-Tdp-43∆NLS mice

**Fig. 7:**
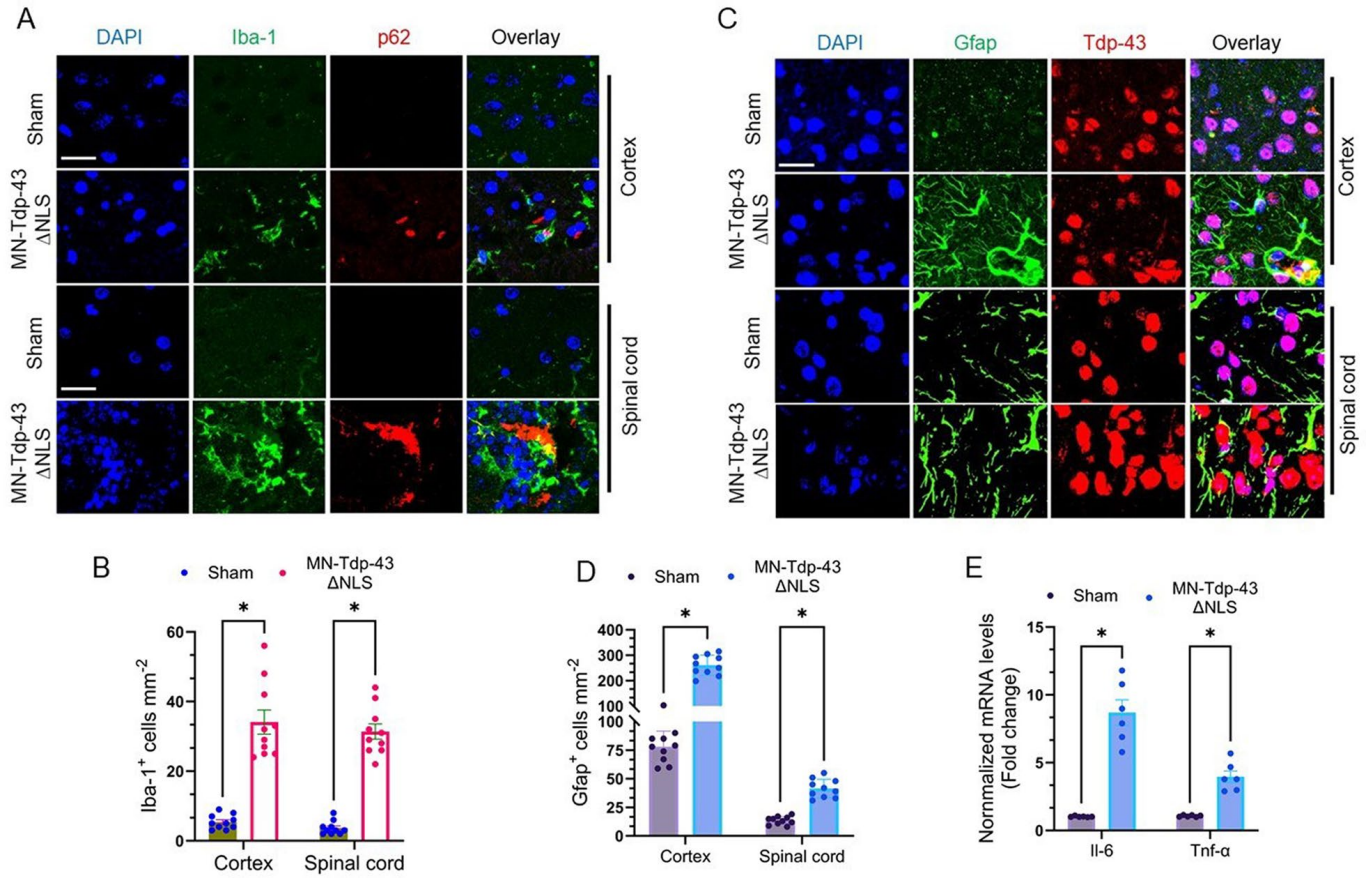
Tdp-43∆NLS mice display Tdp-43 aggregation-induced neuro-inflammation in the CNS. (**A-B**) Representative IF colocalization images showing an increased population of Iba-1^+^ (Green) activated microglia surrounding p62^+^ cells (neurons; Red) in the brain cortex and spinal cord tissues in MN-Tdp-43∆NLS mice compared to sham mice (N = 10 different 1mm^2^ microscopic fields from 6 mice/group). Nuclei were counterstained with DAPI. Scale bar = 10 µm (**A**). (**B**) Quantitation of the number of Iba-1^+^ cells per mm^2^. (**C-D**) Representative IF colocalization images displaying activated Gfap^+^ astrocytes surrounding neurons with Tdp-43 pathology in the cortical region of MN-Tdp-43∆NLS mice but not in sham mice. Nuclei were counterstained with DAPI. Scale bar = 10 µm (**C**). (**D**) Quantitation of the number of Gfap^+^ cells per mm^2^ in the cortex and spinal cord. (**E**) Quantitation of relative mRNA levels (fold change) of neuro-inflammatory markers Il-6 and Tnf-α in the cortical tissues of MN-Tdp-43∆NLS and sham mice (N = 6/group). Gapdh served as the internal control. Data are expressed as mean ± SEM and analyzed by multiple paired t-tests. *, *P* < 0.05

Previous studies suggest that protein aggregation pathology induces hyperactivation of the neuro-inflammatory responses in the CNS of ALS patients [53, 54]. To examine the extent of neuroinflammation associated with Tdp-43 pathology, we performed IHC staining using anti-p62 (protein aggregation marker) and Iba-1 (activated microglial marker) antibodies in the cortical and spinal cord tissue sections of MN-Tdp-43∆NLS and sham mice (Fig. 7A). Interestingly, we found that both cortex and spinal cord regions in the CNS had significantly higher (cortex: -28.70 ± 3.350, *P* = 0.000013; spinal cord: -27.70 ± 2.399, *P* = 0.000002) populations of activated microglia in the vicinity of strong p62 signals in MN-Tdp-43∆NLS mice than in respective sham controls (Fig. 7B). Further, we assessed the status of astrocyte activation in MN-Tdp-43∆NLS mice in comparison to sham mice by IHC staining with anti-Tdp-43 and Gfap antibodies (Fig. 7C), demonstrating significantly higher populations of activated astrocytes in the cortex (-182.9 ± 14.00; *P* < 0.000001) and spinal cord (-28.00 ± 3.102; *P* = 0.000008) of MN-Tdp-43∆NLS mice than sham mice (Fig. 7D) and that activated Gfap-positive astrocytes wrapped the neurons with mislocalized Tdp-43 pathology in the CNS (Fig. 7C), suggesting that endogenous Tdp-43 mislocalization-associated protein aggregation can induce the inflammatory pathway and signal brain-resident immune cells to initiate immune stress response. The qRT-PCR analysis also showed significantly increased expressions of inflammatory markers such as Il-6 (8.48-fold; *P* = 0.000872) and Tnf-α (3.71-fold; *P* = 0.000988) in the brains of MN-Tdp-43∆NLS mice compared to sham controls (Fig. 7E).

**Fig. 8:**
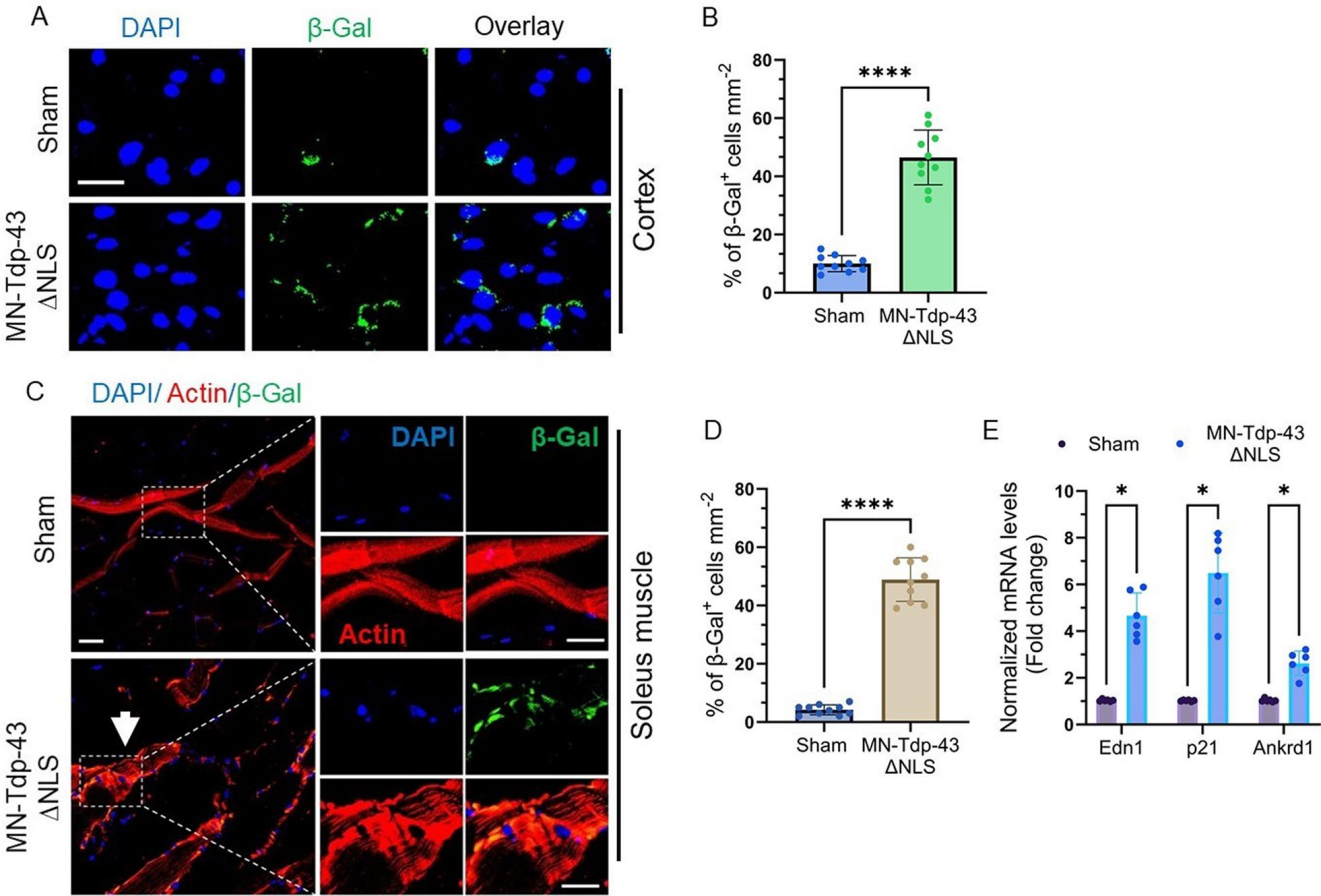
Tdp-43∆NLS mice exhibit neuronal senescence phenotype in the CNS and hind-limb muscle. (**A-B**) Representative IF images with fluorescence-based (β-Gal) senescence staining in the cortex of sham and MN-Tdp-43∆NLS mice (N = 6/group). Nuclei were counterstained with DAPI. Scale bar = 10 µm (**A**). (**B**) Quantitation of percent of β-Gal positive senescent cells using paired t-test. ****, *P* < 0.0001. (**C-D**) IF images of hind-limb soleus muscle tissues stained with anti-Actin (Alexa-Fluor 647) and fluorescent β-Gal (488 nm) from sham and MN-Tdp-43∆NLS mice. The white arrow indicates defective actin polymerization in the soleus muscle of MN-Tdp-43∆NLS mice. Nuclei were counterstained with DAPI. Scale bars = 20 µm and 10 µm (inset images) (**C**). (**D**) Quantitation of percent of β-Gal positive senescent cells using paired t-test. ****, *P* < 0.0001. (**E**) Quantitation of relative mRNA levels (fold change) of senescence-associated markers Edn1, p21, and Ankrd1 in cortical tissues of sham and MN-Tdp-43∆NLS mice (N = 6/group). Gapdh served as the internal control. Data are expressed as mean ± SEM and analyzed by multiple paired t-tests. *, *P* < 0.05

Notably, recent studies have linked the neuronal senescence phenotype to neuroinflammatory conditions in several neurodegenerative diseases [55, 56]. Moreover, DNA damage has been implicated in precipitating acute cellular senescence, independent of telomere shortening [57]. Hence, we sought to investigate the association of Tdp-43 pathology-induced genome damage and senescence in this MN-Tdp-43∆NLS mouse model. In this context, we conducted senescence analysis in both the cortex and hindlimb soleus muscle tissues that showed the denervation pathology using a fluorophore-tagged β-Gal reagent (Fig. 8A). The staining results revealed that MN-Tdp-43∆NLS mice brain cortex (layers III-IV) had a significantly higher percentage of β-Gal-positive senescent cells (36.50 ± 3.110%; *P* < 0.0001) than the sham mice (Fig. 8B). In the case of muscle tissues, the β-Gal staining in combination with anti-actin antibody demonstrated disorganization of actin fiber bundles (Fig. 8C), compared to prominent actin bundles in sham, along with a significantly increased (44.70 ± 2.530%; *P* < 0.0001) population of senescent muscle cells in the hindlimb soleus tissue of MN-Tdp-43∆NLS mice (Fig. 8D). Additionally, qRT-PCR analysis of senescence-associated marker genes showed several folds higher expressions of *Edn1* (4.5-fold; *P* = 0.000657), *p21* (6.35-fold; *P* = 0.001106) and *Ankrd1* (2.54-fold; *P* = 0.001106) in MN-Tdp-43∆NLS mice than in sham controls (Fig. 8E).. Together, these results suggest that the targeted expression of Tdp-43∆NLS variant in the MN induces physiological DNA DSB repair inhibition, leading to persistent inflammation and senescence-mediated loss of neurons in the CNS under Tdp-43 pathological conditions.

## Discussion

TDP-43 mislocalization and aggregation are key features observed in a majority of ALS cases and approximately 40% of FTD patients [1, 58, 59]. The neuropathological and clinical findings indicate that overlapping pathogenic mechanisms involving TDP-43 proteinopathies contribute to neurodegeneration [60–62]. Nevertheless, reproducing human ALS/FTD symptoms in rodent models has been challenging due to TDP-43’s complex regulation and sensitivity to dosage changes [42, 63].

In our initial cell model experiments, we observed that even partial nuclear clearance of TDP-43 was sufficient to disrupt the balance of endogenous DNA damage and repair, leading to the accumulation of unrepaired DNA breaks in the nuclear genome. While Winton et al. 2008 [64] previously reported correlation of deletion of the NLS sequence in TDP-43 with its enhanced tendency for mislocalization and aggregation, our study is the first to establish the connection between TDP-43 mislocalization and spontaneous genome instability in neurons in vivo.

To further investigate TDP-43 mislocalization pathologies and related motor function defects in vivo, we generated a novel endogenous CRISPR KI Tdp-43∆NLS mouse model conditionally expressing a murine Tdp-43 variant lacking NLS (82–98 aa) sequence. This model uniquely mimics the early stages of ALS, showing Tdp-43 mislocalization and aggregation as well as progressive neurodegeneration, the key pathological features of disease progression. Our model eliminates the limitations of previous models, such as the rapid onset of aberrant motor symptoms and phenotypic artifacts from constitutive transgene overexpression.

We utilized the FLEX-based activation to target the *Tardbp* allele, employing Cre driver-mediated recombination of loxP-loxP or mutant loxM-loxM sites. This design ensures control over the recombinase reaction in differentiated or mature cell types, preventing Cre-loxP-mediated aberrant chromosomal rearrangements and loss of the target allele in embryonic stem cells [65]. Our endogenous KI model is thus novel and distinct from previous ALS-TDP-43 overexpression and downregulation mice models [27, 45, 46, 66–68], and NLS-deleted Tdp-43 variant from the murine *Tardbp* gene’s locus is expressed, specifically in MNs. This approach eliminates the potential complications of transgene insertion and the development of aggressive motor phenotypes, which rarely mimic human ALS pathophysiology.

To recapitulate the disease progression from pre-symptomatic to symptomatic stages, we incorporated a hemizygous bigenic MN-specific Tdp-43∆NLS allele in most studies. The bigenic Cre::Tdp-43∆NLS mice suffered from progressive motor dysfunctions, gait asymmetry, early-stage myogenic ALS pathology, and MN degeneration, simultaneously, pTDP‐43- and ubiquitin‐positive pathology in the dorsolateral and dorsoventral spinal cord, reflected early ALS symptoms [69]. This progression is more nuanced compared to milder phenotypes of neuromuscular abnormalities seen in mice with mutations in Fus, Vcp, and Sod1 [70–72]. At around 12 months of age (equivalent to 45–50 years of human age) [73], these mice progressively developed signs of gait disorders and mild clasping symptoms in their hind limbs, without paralysis or premature death.

Furthermore, our mouse model is the first to demonstrate the link between Tdp-43 pathology and induction of DNA break accumulation, resulting in enhanced neuroinflammatory responses in the CNS. Using a combination of cellular, molecular, and histopathological readouts, along with in vivo motor function tests, we show that aberrant mislocalization of Tdp-43∆NLS variant and its subsequent aggregation recapitulate the key pathologic features of ALS-TDP-43. Our analysis also revealed potential crosstalk among proteinopathy, genome damage, neuroinflammation, and neuronal senescence in this early symptomatic Tdp-43∆NLS model of ALS.

While accumulating evidence underscores a critical connection between genome damage and neuron loss in ALS-TDP-43 and related diseases, to date, only rNLS8 (hTDP-43∆NLS transgenic) line has shown approximately 2–3 folds overexpression of DNA damage-inducible transcript 3 (Chop), and growth arrest and DNA-damage-inducible 45 gamma (Gadd45γ), as the early markers of cellular stress and death [74–76]. However, the perturbed DNA damage and repair response pathways were not tested in TDP-43 pathology-afflicted neurons. Hence, we tested this in our endogenous MN-Tdp-43∆NLS mouse model and revealed in the CNS (cortex and spinal cord) that nuclear clearance and subsequent pathological aggregation of Tdp-43 in MN-specific manner led to the accumulation of DNA break foci (Fig. 5C-F), hyperactivation of neuroinflammatory factors such as Iba-1, Il-6, and Tnf-α (Fig. 7), resulting in the manifestation of motor deficit phenotypes in 12 months age. We also recapitulated our initial finding that aggregated mutant TDP-43 can trap DNA repair factors in the cytosol [21], thereby preventing their nuclear translocation in response to genome damage and inhibiting DNA repair processes. Notably, Tdp-43∆NLS expression-induced Tdp-43 mislocalization into the cytosol was correlated with loss of Map2 in the cortex (Fig. 3A and supplemental Fig. 1C) and spinal cord (data not shown), consistent with previous reports [77]. Although GFAP-positive astrocytes were activated or accumulated in the vicinity of damaged neurons with Tdp-43 proteinopathies (Fig. 7C), these astrocytes did not exhibit any Tdp-43 protein mislocalization or aggregation phenotypes, suggesting that observed motor phenotypes were specifically caused by MN-specific Tdp-43 proteinopathy in MN-Tdp-43∆NLS mice. Furthermore, our Tdp-43∆NLS mouse is unique in terms of avoiding non-specific interaction between hTDP-43∆NLS and cryptic exon splicing of murine transcripts, for example, *Stmn2*, *Unc13a* and *Nptx2* genes, due to differences in RNA sequences at target binding sites [52, 78–80]. Besides, mice models overexpressing ALS-linked TDP-43 mutants, such as Q331K [66, 81–83], are likely to involve specific dysregulation in RNA splicing complex formation due to perturbed binding of hnRNP factors – hnRNPA1, hnRNPA2/B1, E2 [39, 84, 85] at the C-terminal prion-like domain of TDP-43, instead of general TDP-43 aggregation pathobiology. Our Tdp-43∆NLS can induce both nuclear loss-of-function and cytosolic gain-of-toxicity-related pathological changes in the CNS, closely mimicking human ALS conditions. A comparison of relative advantages and disadvantages among some of the existing ALS/FTD-TDP-43 mice models is presented in Supplemental Table 1.

Further investigation of the effect Tdp-43∆NLS variant at the neuromuscular junctions revealed a prominent loss of muscle fiber integrity and loss of Stmn2 levels in the hindlimb (which showed gait defects) soleus muscle in MN-Tdp-43∆NLS mice compared to age-matched sham mice. More importantly, we found a unique pattern of the largest muscle fiber protein Titin – both overexpression and fragmentation yielding pathological 25 kDa truncated variant. Titin overexpression [86] and fragmentation [87] are pathological for skeletal muscle integrity. Titin pathology has been linked to hereditary myopathy [88] and ALS [40], and its 25 kDa fragment peptide has been proposed as a promising biomarker for ALS patients [89]. We speculate that observed actin bundle disorganization in the skeletal muscle might be linked to TDP-43 pathology-induced titin expression dysregulation and altered sarcomere dynamics [90] in MN-Tdp-43∆NLS mice. Therefore, this mouse model could be useful for screening therapeutics against both CNS and muscle pathologies, like ALS. However, detailed mechanistic investigations are warranted for understanding the actual disease mechanism in the background of TDP-43 pathology.

Our findings also underscore the potential role of neuronal senescence related to TDP-43 pathology in neurodegeneration. In MN-Tdp-43ΔNLS mice CNS and hindlimb muscle tissues, we observed a significant increase in the number of senescence-positive cells as well as elevated expression levels of senescence-associated genetic markers (Fig. 8). Furthermore, a clear overlap of senescence staining and disorganized actin polymer bundles in limb muscle suggest crucial role of senescence mechanism in muscle atrophy in TDP-43 pathology. This observation is important in the sense that we don’t know yet how these senescent cells would influence the progression of the disease and whether these cells could be rescued by mitigating their DNA repair defects through targeted therapeutics. Although early senescence may confer protection to neurons against lethal damage [91–93], emerging evidence suggests that neuronal senescence plays a pivotal role in neuron loss, resulting in motor and cognitive dysfunctions in ALS/FTD [94–96]. Furthermore, there is an important crosstalk between cellular senescence and neuroinflammation. C-X-C motif chemokine receptor 2 (CXCR2) was found to increase significantly triggering neuronal apoptosis in sporadic ALS [97]. On the other hand, senescent cells activate a CXCR2-mediated self-amplifying secretory network which promotes growth arrest [98]. In this study, our results indicate possible crosstalk among Tdp-43 mislocalization, increased DNA damage, and neuronal senescent cells in MN-Tdp-43∆NLS mice brain and spinal cord regions. Future research should focus on dissecting the molecular characteristics of these senescent motor neurons and exploring DNA repair-targeted therapies for ALS, FTD, and related neurodegenerative conditions.

Our ALS-Tdp-43 mouse model demonstrates the clear manifestation of key pathological hallmarks of ALS/FTD at the molecular level while maintaining a non-paralytic motor deficit condition. As such, it can offer investigators unique opportunities to decipher the disease-causing early-stage pathogenic mechanisms in MNs that might be reverted by therapeutic drugs, even in long-term treatments – a condition that is difficult to sustain in other aggressive disease models.

In conclusion, multiple animal models for ALS/FTD are obligatory for a comprehensive understanding of their complex and progressive pathogenesis. Such models, including those based on overexpression or knockdown of critical genes, play a crucial role in unraveling specific etiological functions and/or toxicity of the disease-related proteins. Our model, uniquely replicating both the nuclear loss of Tdp-43 and its cytosolic aggregation – two hallmark features of ALS/FTD – adds a new dimension to the existing array of animal models. This model not only mirrors key disease mechanisms, including protein mislocalization, aggregation, and markers of TDP-43’s pathological forms but also genome instability, inflammation, senescence, and neuronal dysfunction, along with gait abnormalities. Furthermore, this model offers the opportunity to investigate the very early-stage etiological factors of ALS and the concomitant impact of aging on ALS progression. Such comprehensive representation will make this model an invaluable tool for testing new therapeutic concepts and deepening our understanding of these debilitating neurodegenerative diseases.

## Supplementary Information

Additional file 1 .

Additional file 2.

## Data Availability

All relevant data generated and analyzed in this study are available in this manuscript, online supplementary information, or upon reasonable request.
