# Supplementary material for: Endogenous TDP-43 mislocalization in a novel knock-in mouse model reveals DNA repair impairment, inflammation, and neuronal senescence
Source: Acta Neuropathol Commun. 2025 Mar 8;13:54. doi: 10.1186/s40478-025-01962-9 (PMC11889789; doi:10.1186/s40478-025-01962-9)
Supplement: Supplementary file 1 — Additional file 1 . [file 40478_2025_1962_MOESM1_ESM.pdf]

Supplemental Materials

Endogenous TDP-43 mislocalization in a novel knock-in mouse model reveals DNA repair impairment, inflammation, and neuronal senescence

Joy Mitra[1*], Manohar Kodavati[1], Prakash Dharmalingam[1], Erika N. Guerrero[1,†], K. S. Rao[2], Ralph M. Garruto[3,4], Muralidhar L. Hegde[1,5,*]

[1] Division of DNA Repair Research, Center for Neuroregeneration, Department of Neurosurgery, Houston Methodist Research Institute, Houston, TX 77030, USA

[2] Department of Biotechnology, Koneru Lakshmaiah Education Foundation Deemed to be University, Green Fields, Vaddeswaram, Andhra Pradesh 522502, India

[3] Department of Anthropology, Binghamton University, State University of New York, Binghamton, NY 13902, USA

[4] Department of Biological Sciences, Binghamton University, State University of New York, Binghamton, NY 13902, USA

[5] Department of Neuroscience, Weill Cornell Medical College, New York, NY 10065, USA

*Corresponding authors: J.M. (jmitra@houstonmethodist.org) and M.L.H. (mlhegde@houstonmethodist.org)

Present affiliations: [†] Gorgas Memorial Institute for Health Studies, Avenida Justo Arosemena y Calle 35, Panama City, Republic of Panama & Sistema Nacional de Investigación, SENACYT, Panama City, Republic of Panama

Supplemental materials include one table and its related references, and five figures along with figure legends.

Supplemental **Table 1**: A comparison of TDP-43 pathologies, disease phenotypes and practical applications of various types of TDP-43 mouse models of ALS and FTD.

Model Type	TDP-43 variant	Disease Phenotypes	Advantages	Disadvantages	Reference
Cisgenic – Knock-in	Tdp-43ΔE2/3	Spinal motor neuron-specific Tdp-43ΔE2/3 nuclear depletion-linked ALS-like phenotypes	- Allows to study motor neuron-specific Tdp-43 pathologies and motor defects.<br>- Tdp-43 pathology-induced astrocytosis.	- Not suitable for studying ALS/FTD-TDP-43 related RNA processing and misfolded protein clearance mechanisms.<br>- Posses disease-irrelevant Tdp-43 mutation.<br>- Pathological effect of Tdp-43ΔE2/3 variant in the motor cortex is unknown.<br>- Not suitable for studying long-term age-associated ALS phenotypes.	[1]
Transgenic	rNLS (hTDP-43ΔNLS)	Insoluble phosphorylated cytoplasmic TDP-43 aggregates in the CNS, brain and muscle atrophy, leading to death.	- Allows rapid onset ALS pathology.<br>- Presents most ALS-like symptoms upon hTDP-43ΔNLS induction.<br>- Presents aggressive but reversible motor deficits.	- hTDP-43ΔNLS-mediated nuclear loss of wildtype murine Tdp-43.<br>- Non-specific interaction between hTDP-43ΔNLS and murine mRNA, resulting in aberrant alternative splicing.<br>- Aggressive model of ALS, not suitable for pre-symptomatic biomarker discovery for early detection.<br>- The underlying mechanism of reversible motor phenotype is unclear.	[2]
Cisgenic – Knock-in	Mnx1-Tdp-43ΔNLS	Nuclear clearance and cytoplasmic-aggregation of Tdp-43ΔNLS in motor neurons and associated ALS-type motor defects and muscle weakness	- Suitable for investigating progressive motor neuron degeneration mimicking ALS.<br>- Allows cell type-specific and conditional expression of Tdp-43ΔNLS mutant, with otherwise intact RRM domains.<br>- Suitable for pre-symptomatic pathomechanistic discoveries and biomarker development.<br>- Suitable for studying the gradual nuclear loss of Tdp-43ΔNLS and altered mRNA processing and expression.<br>- Suitable for investigating Tdp-43 pathology-linked genome instability, inflammation, and neuronal senescence pathways for therapy development.	- Disease onset and manifestation are slow (10-12 months).<br>- Not suitable for aggressive ALS pathology studies.	This study.

Transgenic	hTDP-43 WT	hTDP-43 overexpression-induced TDP-43 ubiquitination, phosphorylation and fragmentation pathologies. Moderate to aggressive ALS/FTD phenotypes.	-Allows CNS-targeted TDP-43 WT overexpression-associated disease phenotypes.	- Not suitable for examining motor neuron-specific TDP-43 proteinopathies.<br>- Disease onset and aggressiveness vary widely from mild to severe across the transgenic subtypes.<br>-Not reliable for ALS/FTD-TDP-43-linked RNA alternative splicing and biomarker discovery studies.	[3]
Cisgenic – knock-in	Tdp-43 Q331K	Sporadic ALS-Tdp-43 Q331K variant-induced Tdp-43 gain-of-function phenotypes.	- Suitable for studying genetic causes of male dominance in ALS/FTD.<br>- Allows investigating Tdp-43 Q331K-related aberrant mRNA splicing.<br>- Enables investigating Tdp-43 gain-of-function motor and behavioral phenotypes for Q331K mutant.<br>- Allows investigating Tdp-43 pathology-induced metabolic	- Not suitable for investigating TDP-43 loss-of-function and gain-of-toxicity phenotypes.<br>- A rare mutation model.<br>- Lacks ALS-like limb muscle pathologies.	[4]
Transgenic	hTDP-43 A315T	TDP-43-mediated neurodegeneration in the brain and spinal cord without cytosolic aggregation of TDP-43	Allows studying<br>- ALS-like motor and pathological phenotypes in a short span of time.<br>- hTDP-43 overexpression-induced disease mechanism in mice.	- Non-specific TDP-43 pathology for motor neurons and motor cortex.<br>- High levels (3-fold) of Tg-TDP-43 overexpression.<br>- Early onset (1-2 months age) pathological symptoms resembling advanced stages of ALS/FTD.<br>- Possibility of aberrant interactions between Tg-TDP-43 A315T and endogenous mRNA and Tdp-43.<br>- Does not recapitulate human ALS aggregation phenotypes.	[5]

ALS: amyotrophic lateral sclerosis; FTD: frontotemporal dementia; E2/3: Exon 2 and Exon 3; NLS: nuclear localization signal; CNS: central nervous system; Tg: transgenic.

Reference

1. Wu LS, Cheng WC, Shen CK (2012) Targeted depletion of TDP-43 expression in the spinal cord motor neurons leads to the development of amyotrophic lateral sclerosis-like phenotypes in mice. J Biol Chem 287:27335-27344. https://10.1074/jbc.M112.359000
2. Walker AK, Spiller KJ, Ge G, Zheng A, Xu Y, Zhou M, Tripathy K, Kwong LK, Trojanowski JQ, Lee VM (2015) Functional recovery in new mouse models of ALS/FTLD after clearance of pathological cytoplasmic TDP-43. Acta Neuropathol 130:643-660. https://10.1007/s00401-015-1460-x

3. Wils H, Kleinberger G, Janssens J, Pereson S, Joris G, Cuijt I, Smits V, Ceuterick-de Groote C, Van Broeckhoven C, Kumar-Singh S (2010) TDP-43 transgenic mice develop spastic paralysis and neuronal inclusions characteristic of ALS and frontotemporal lobar degeneration. Proc Natl Acad Sci U S A 107:3858-3863. https://10.1073/pnas.0912417107
4. White MA, Kim E, Duffy A, Adalbert R, Phillips BU, Peters OM, Stephenson J, Yang S, Massenzio F, Lin Z *et al* (2018) TDP-43 gains function due to perturbed autoregulation in a Tardbp knock-in mouse model of ALS-FTD. Nat Neurosci 21:552-563. https://10.1038/s41593-018-0113-5
5. Wegorzewska I, Bell S, Cairns NJ, Miller TM, Baloh RH (2009) TDP-43 mutant transgenic mice develop features of ALS and frontotemporal lobar degeneration. Proc Natl Acad Sci U S A 106:18809-18814. https://10.1073/pnas.0908767106

Supplemental Fig. 1

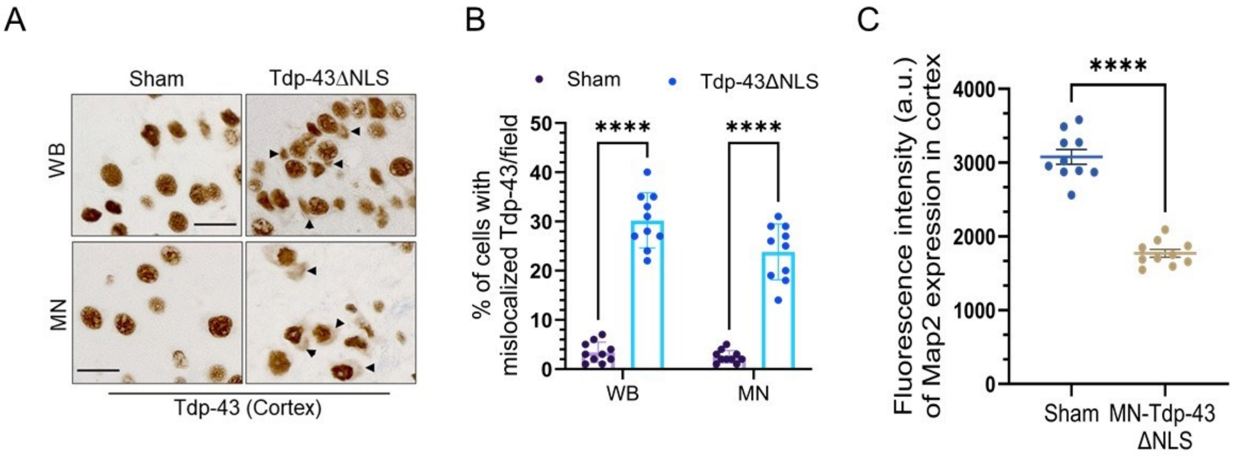

Supplemental Fig. 1. (Related to **Fig. 3**) **Immunohistochemical analysis of Tdp-43 expression phenotypes in different Tdp-43ΔNLS mice models**. (**A-B**) Immunohistochemistry (IHC) with anti-TDP-43 antibody in the motor cortex of ALS mice Ubc-Cre::Tdp-43ΔNLS [whole body (WB)-Tdp-43ΔNLS hence after; *upper panel*] and Mnx1-Cre::Tdp-43ΔNLS [motor neuron (MN)-Tdp-43ΔNLS hence after; *lower panel*]. Scale bar = 20 μm (**A**). (**B**) Quantitation of cells with Tdp-43 mislocalization phenotype in sham versus WB- or MN-Tdp-43ΔNLS mice cortical tissues (N = 6 mice/group) by two-way ANOVA. ****, $P < 0.0001$. (**C**) Quantitation of fluorescence intensity (arbitrary unit, a.u.) of Map2 levels in Layers III-IV of the cortex of sham and MN-Tdp-43ΔNLS mice. Data are expressed as mean ± SEM and analyzed by t-test. ****, $P < 0.0001$.

Supplemental Fig. 2

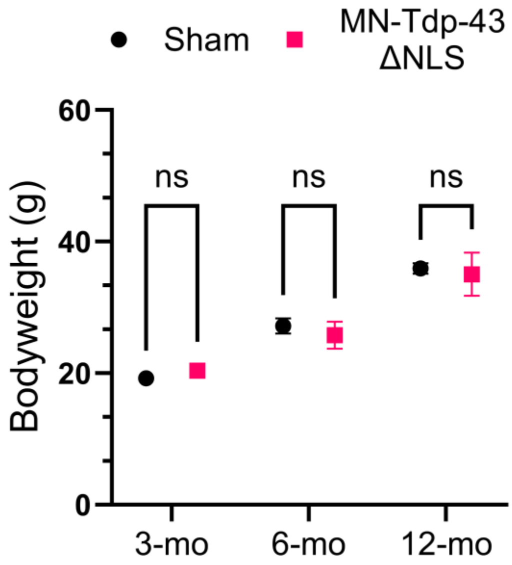

Supplemental Fig. 2. Assessment of bodyweight at different developmental stages. Quantitation of bodyweight of Mnx1-Cre::Tdp-43ΔNLS (MN-Tdp-43ΔNLS) mice**.** Data are expressed as mean ± SEM and analyzed by two-way ANOVA. N = 6 per group. ns = non-significant.

Supplemental Fig. 3

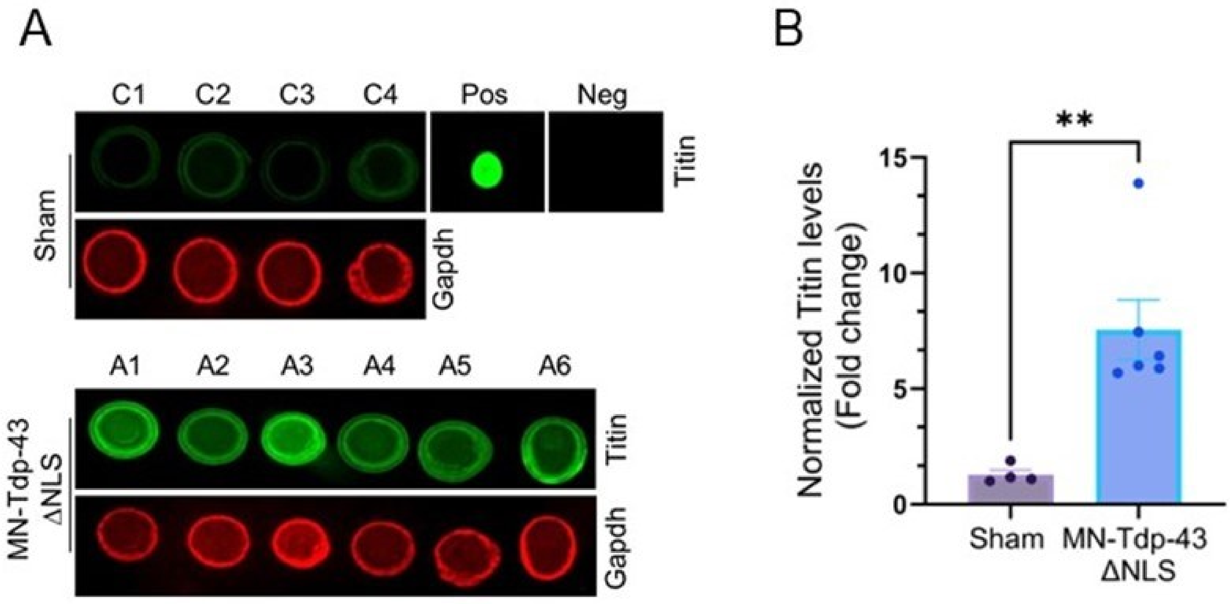

Supplemental Fig. 3 (Related to **Fig. 5**). **Motor neuron (MN)-specific Tdp-43∆NLS expression causes muscle atrophy and gait deficits in Tdp-43 mutant mice.** (**A-B**) Dot blot analysis of Titin (N-terminal fragment) in soleus muscle tissues of MN-Tdp-43ΔNLS (N = 6; A1 – A6) and sham (N = 4; C1 – C4) mice. Gapdh levels were used as the loading controls. Anti-Titin antibody was used as the Positive (Pos) control and lysis buffer as the Negative control (Neg) (**A**). (**B**) Quantitation of normalized mouse Titin levels in two groups. Data are expressed as mean ± SD and analyzed by multiple t-tests. **, $P < 0.01$.

Supplemental Fig. 4

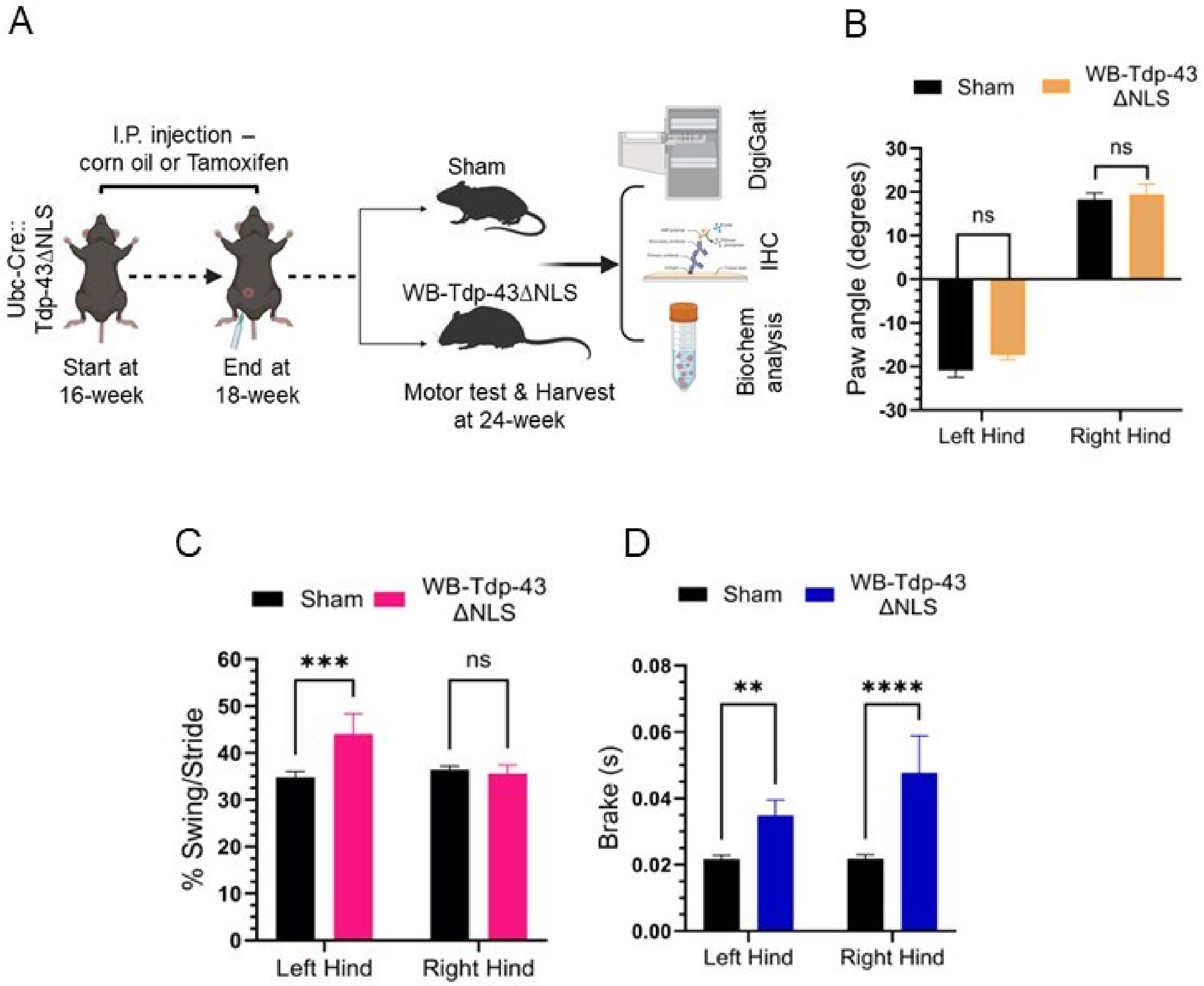

Supplemental Fig. 4 (Related to **Fig. 5**). **Motor function analysis in whole body (WB) Tdp-43ΔNLS expressing mice**. (**A-D**) Schematic illustration of vehicle (corn oil) in sham or tamoxifen treatment in WB-Tdp-43ΔNLS male mice by intraperitoneal (i.p.) administration at 16-week age at a dose of 75 mg/kg every other day for two consecutive weeks (18 weeks), and analysis of motor function at 24-week and harvest for histopathological and biochemical analyses (**A**). DigiGait analysis of paw angle (degrees) (**B)**, percent (%) swing/stride (**C**), and brake duration (sec) (**D**). Data are expressed as mean ± SD and analyzed by two-way ANOVA. N = 6 per group. ns = non-significant; **, $P < 0.01$; ***, $P < 0.001$; ****, $P < 0.0001$.

Supplemental Fig. 5

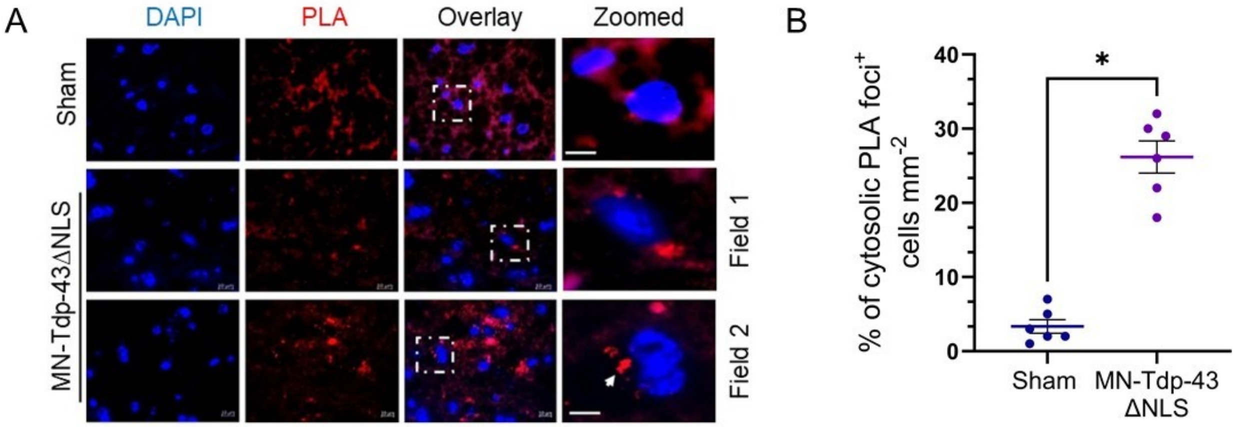

Supplemental Fig. 5 (Related to **Fig. 7**). **MN-Tdp-43ΔNLS mice present the cytosolic sequestration of DNA ligase 4 by Tdp-43ΔNLS.** (**A-B**) Proximity ligation assay (PLA) exhibiting pathological trapping of murine endogenous DNA ligase 4 by the cytosolic Tdp-43ΔNLS in the spinal cord tissue specimens (Fields 1 & 2), as shown by white arrowheads, compared to sham controls. Nuclei were counterstained with DAPI (**A**). Scale bars = 10 μm. (**B**) Quantitation of percent of cells with cytosolic PLA foci positivity per mm^2 of microscopic area. Data are expressed as mean ± SEM and analyzed by Wilcoxon's t-test. *, $P < 0.05$.
