# Supplementary material for: Endogenous TDP-43 mislocalization in a novel knock-in mouse model reveals DNA repair impairment, inflammation, and neuronal senescence
Source: Acta Neuropathol Commun. 2025 Mar 8;13:54. doi: 10.1186/s40478-025-01962-9 (PMC11889789; doi:10.1186/s40478-025-01962-9)

Supplemental Raw Data File

Endogenous TDP-43 mislocalization in a novel knock-in mouse model reveals DNA repair impairment, inflammation, and neuronal senescence

Joy Mitra[1*], Manohar Kodavati[1], Prakash Dharmalingam[1], Erika N. Guerrero[1,†], K. S. Rao[2], Ralph M. Garruto[3,4], Muralidhar L. Hegde[1,5,*]

[1] Division of DNA Repair Research, Center for Neuroregeneration, Department of Neurosurgery, Houston Methodist Research Institute, Houston, TX 77030, USA

[2] Department of Biotechnology, Koneru Lakshmaiah Education Foundation Deemed to be University, Green Fields, Vaddeswaram, Andhra Pradesh 522502, India

[3] Department of Anthropology, Binghamton University, State University of New York, Binghamton, NY 13902, USA

[4] Department of Biological Sciences, Binghamton University, State University of New York, Binghamton, NY 13902, USA

[5] Department of Neuroscience, Weill Cornell Medical College, New York, NY 10065, USA

*Corresponding authors: J.M. (jmitra@houstonmethodist.org) and M.L.H. (mlhegde@houstonmethodist.org)

Present affiliations: † Gorgas Memorial Institute for Health Studies, Avenida Justo Arosemena y Calle 35, Panama City, Republic of Panama & Sistema Nacional de Investigación, SENACYT, Panama City, Republic of Panama

This file contains uncropped images of gels and blots used in this manuscript.

Related to Fig. 1F

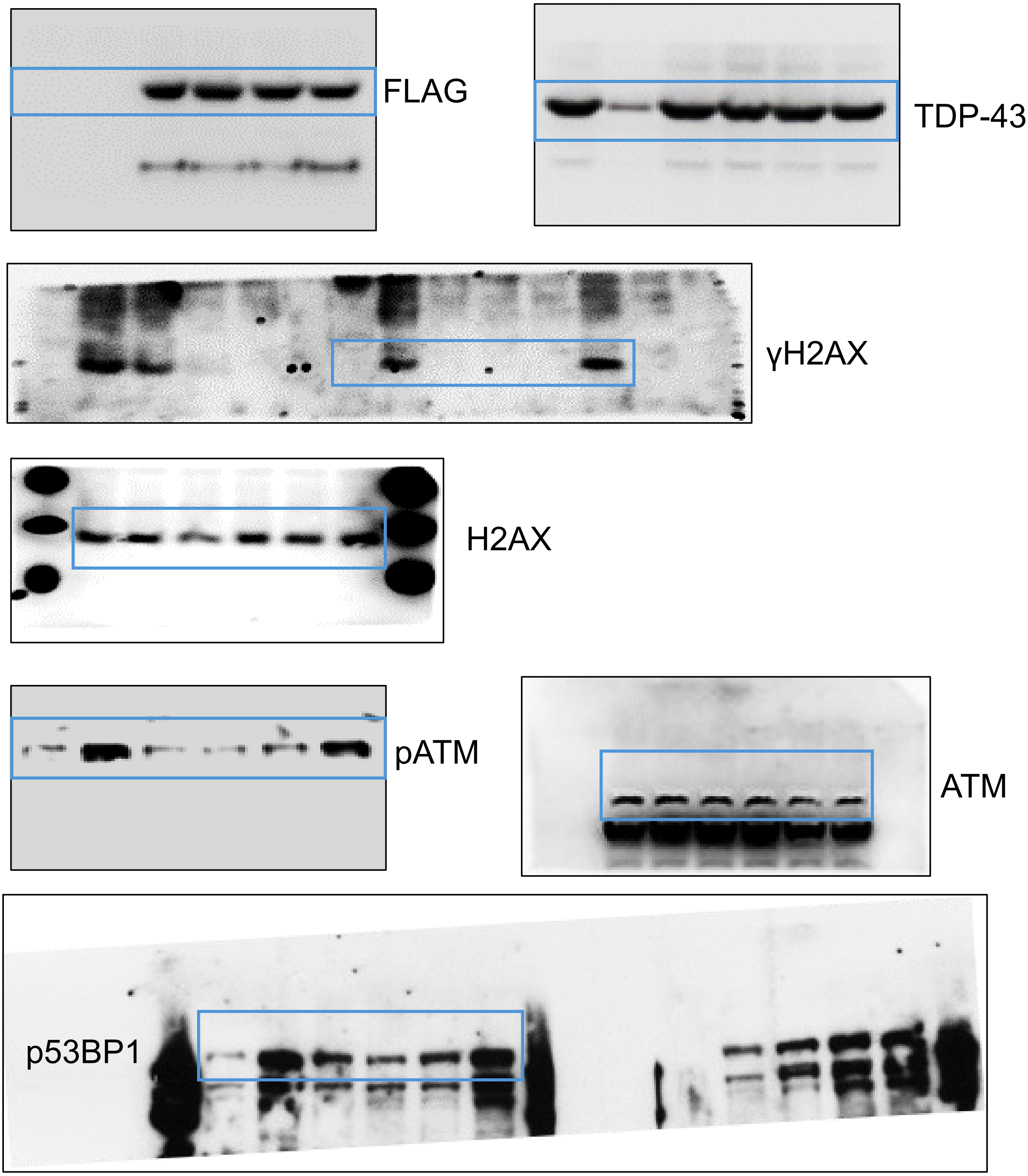

Related to Fig. 1F

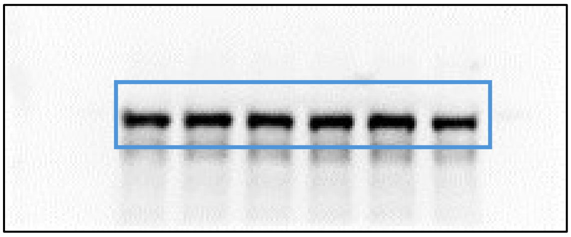

53BP1

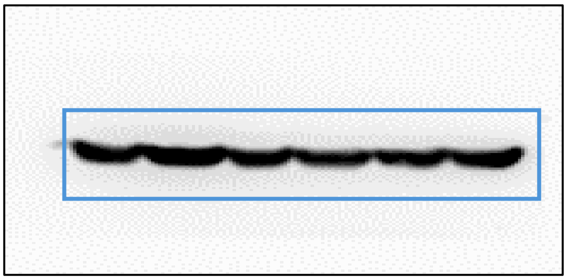

β-actin

Related to Fig. 3I

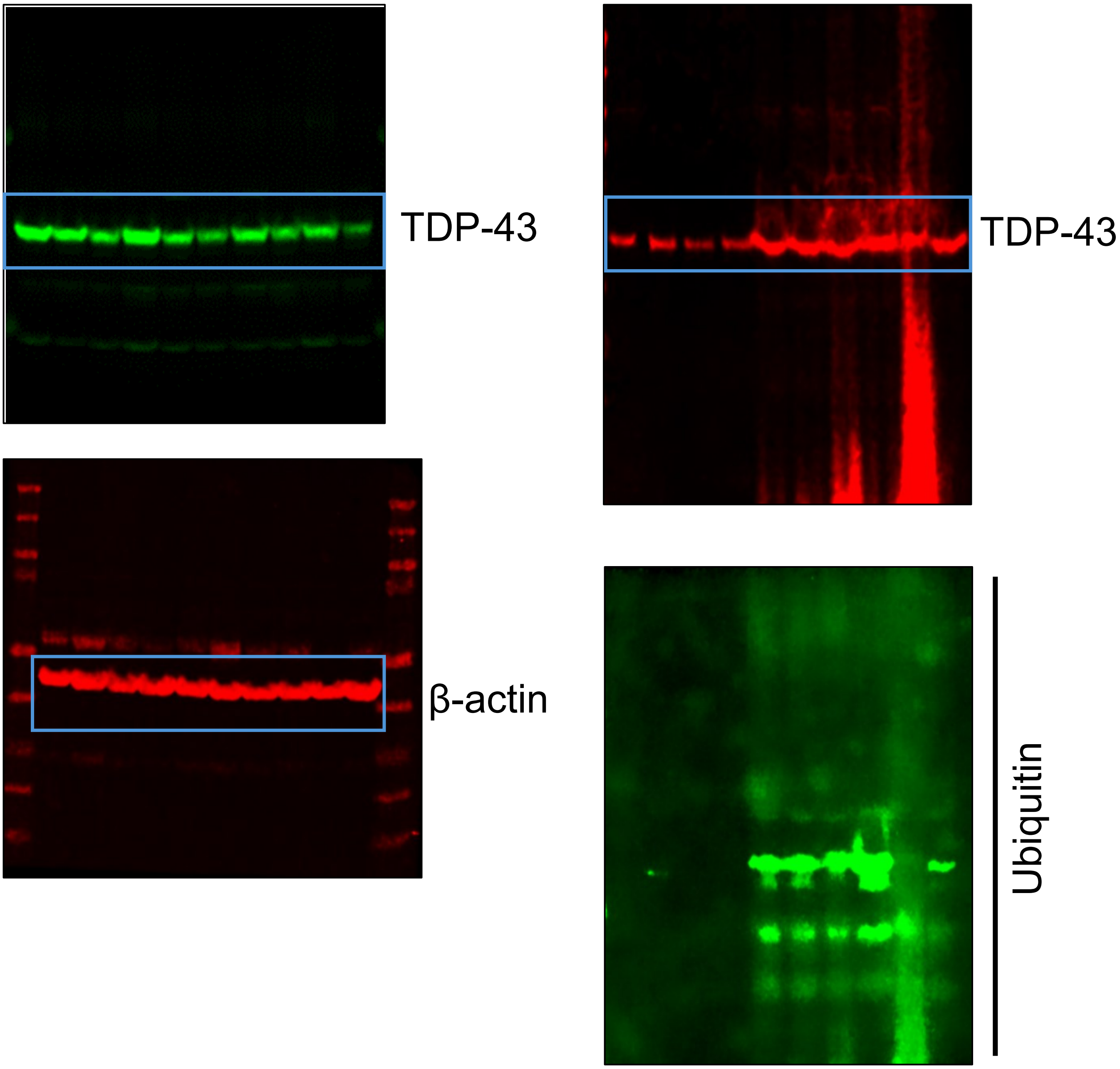

Related to Fig. 4B

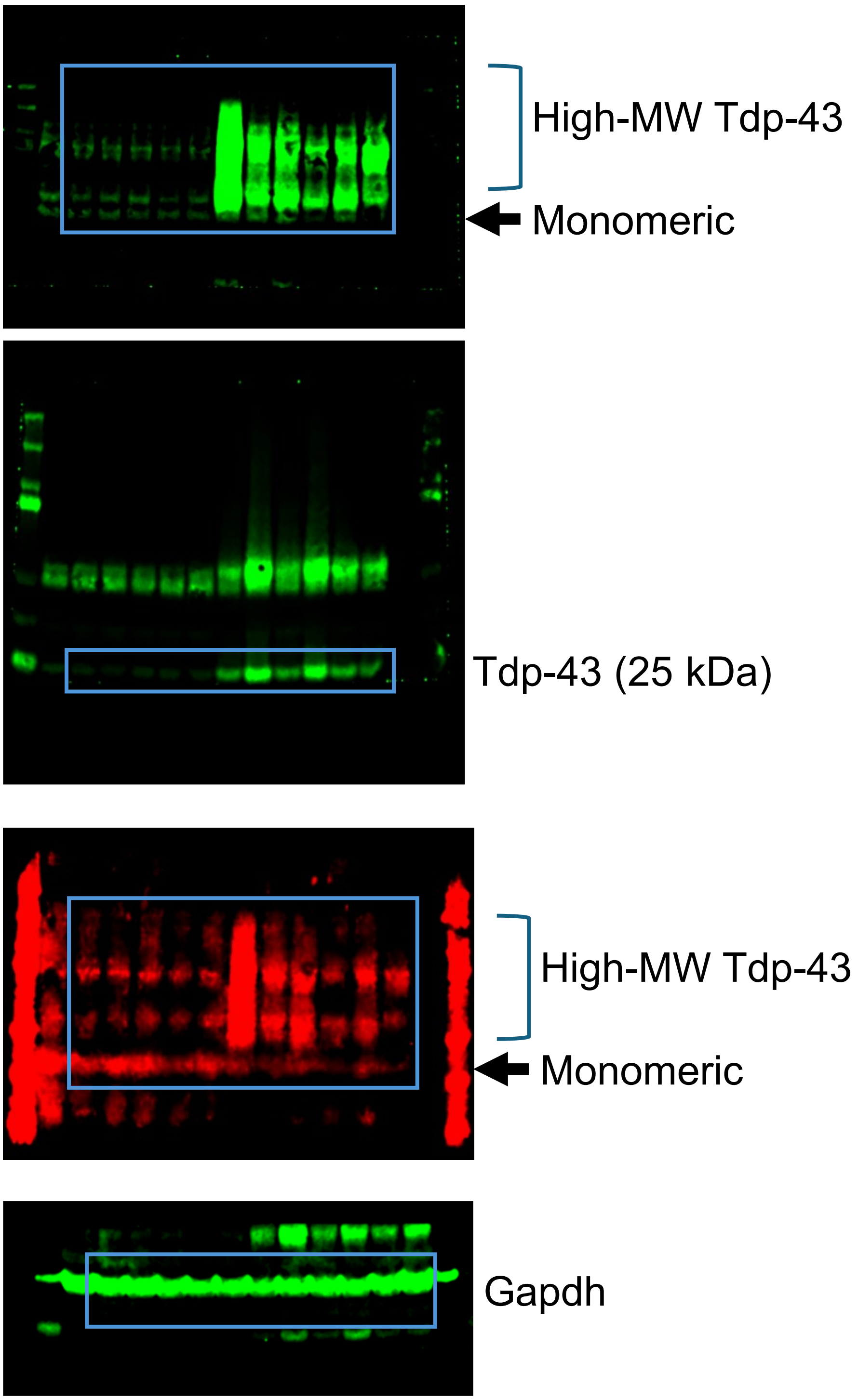

Related to Fig. 4E

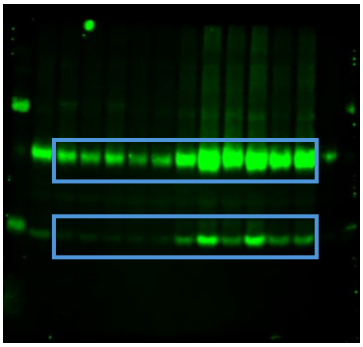

Titin-FL

Titin-Frag.

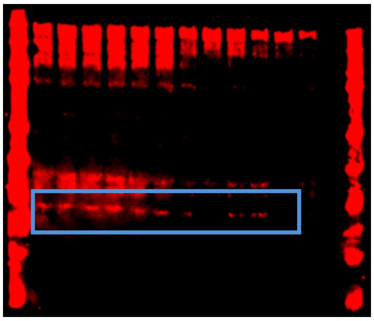

Stmn2

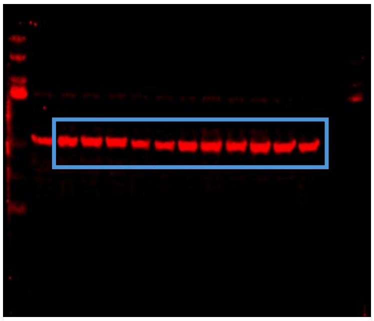

Tubulin

Related to Fig. 5A

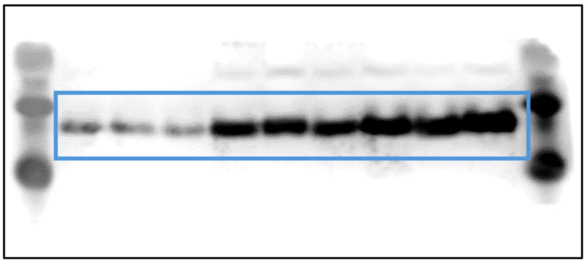

γH2ax

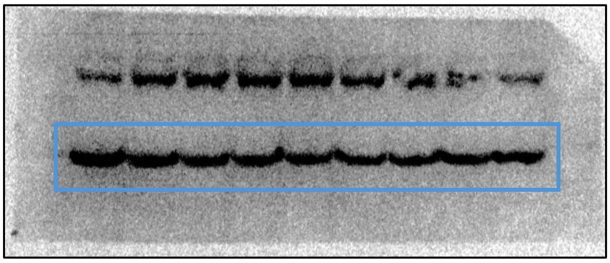

Gapdh

Related to Fig. 5G

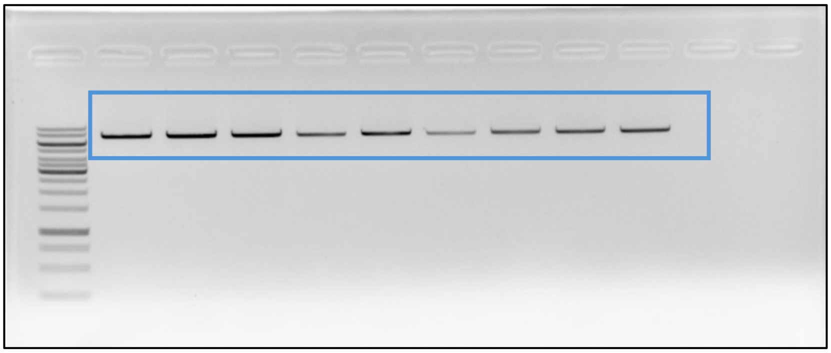

Neurod1

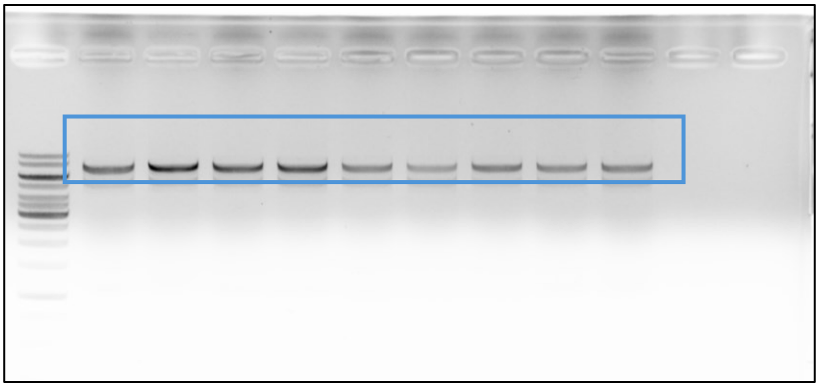

Nanog

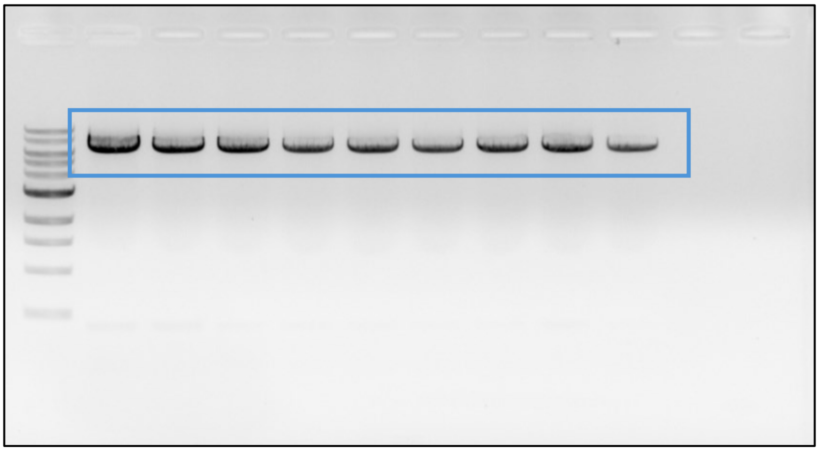

Polβ

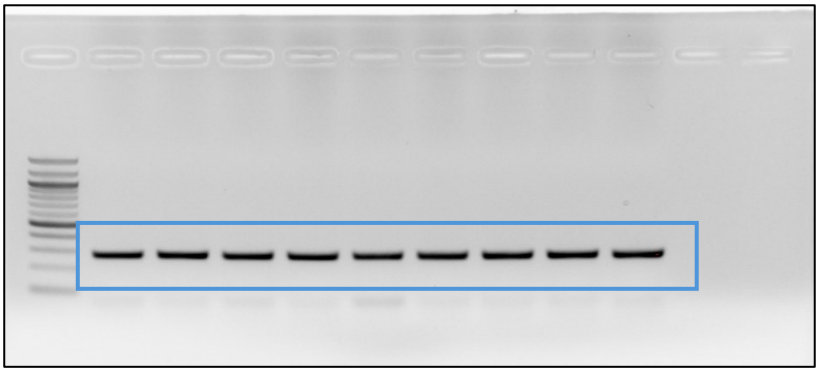

Short amplicon

Related to Supplemental Fig. 3

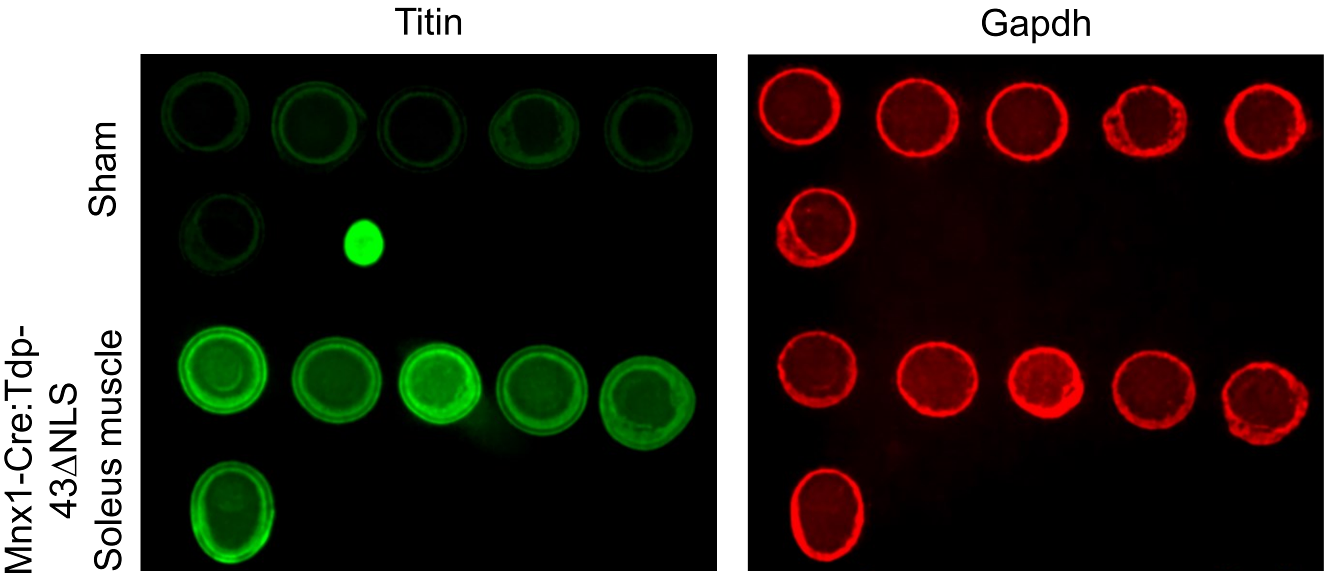

Supplement: Supplementary file 2 — Additional file 2. [file 40478_2025_1962_MOESM2_ESM.pdf]
